# Whole genome sequencing of Canadian *Saccharomyces cerevisiae* strains isolated from spontaneous wine fermentations reveals a new Pacific West Coast Wine clade

**DOI:** 10.1093/g3journal/jkad130

**Published:** 2023-06-12

**Authors:** R Alexander Marr, Jackson Moore, Sean Formby, Jonathan T Martiniuk, Jonah Hamilton, Sneha Ralli, Kishori Konwar, Nisha Rajasundaram, Aria Hahn, Vivien Measday

**Affiliations:** Genome Science and Technology Graduate Program, University of British Columbia, Vancouver, BC V5Z 4S6, Canada; Department of Food Science, Wine Research Centre, Faculty of Land and Food Systems, University of British Columbia, 2205 East Mall, Vancouver, BC V6T 1Z4, Canada; Genome Science and Technology Graduate Program, University of British Columbia, Vancouver, BC V5Z 4S6, Canada; Department of Food Science, Wine Research Centre, Faculty of Land and Food Systems, University of British Columbia, 2205 East Mall, Vancouver, BC V6T 1Z4, Canada; Koonkie Canada Inc., 321 Water Street Suite 501, Vancouver, BC V6B 1B8, Canada; Department of Food Science, Wine Research Centre, Faculty of Land and Food Systems, University of British Columbia, 2205 East Mall, Vancouver, BC V6T 1Z4, Canada; Food Science Graduate Program, Faculty of Land and Food Systems, University of British Columbia, Vancouver, BC V6T 1Z4, Canada; Department of Food Science, Wine Research Centre, Faculty of Land and Food Systems, University of British Columbia, 2205 East Mall, Vancouver, BC V6T 1Z4, Canada; Canada’s Michael Smith Genome Sciences Centre, BC Cancer, 675 West 10th Avenue, Vancouver, BC V5Z 1L3, Canada; Department of Biomedical Physiology and Kinesiology, Simon Fraser University, 8888 University Drive East K9625, Burnaby, BC V5A 1S6, Canada; Koonkie Canada Inc., 321 Water Street Suite 501, Vancouver, BC V6B 1B8, Canada; Koonkie Canada Inc., 321 Water Street Suite 501, Vancouver, BC V6B 1B8, Canada; Koonkie Canada Inc., 321 Water Street Suite 501, Vancouver, BC V6B 1B8, Canada; Department of Food Science, Wine Research Centre, Faculty of Land and Food Systems, University of British Columbia, 2205 East Mall, Vancouver, BC V6T 1Z4, Canada

**Keywords:** yeast, wine, *Saccharomyces cerevisiae*, vineyard, wild, industrial, domesticated, diversity

## Abstract

Vineyards in wine regions around the world are reservoirs of yeast with oenological potential. *Saccharomyces cerevisiae* ferments grape sugars to ethanol and generates flavor and aroma compounds in wine. Wineries place a high-value on identifying yeast native to their region to develop a region-specific wine program. Commercial wine strains are genetically very similar due to a population bottleneck and in-breeding compared to the diversity of *S. cerevisiae* from the wild and other industrial processes. We have isolated and microsatellite-typed hundreds of *S. cerevisiae* strains from spontaneous fermentations of grapes from the Okanagan Valley wine region in British Columbia, Canada. We chose 75 *S. cerevisiae* strains, based on our microsatellite clustering data, for whole genome sequencing using Illumina paired-end reads. Phylogenetic analysis shows that British Columbian *S. cerevisiae* strains cluster into 4 clades: Wine/European, Transpacific Oak, Beer 1/Mixed Origin, and a new clade that we have designated as Pacific West Coast Wine. The Pacific West Coast Wine clade has high nucleotide diversity and shares genomic characteristics with wild North American oak strains but also has gene flow from Wine/European and Ecuadorian clades. We analyzed gene copy number variations to find evidence of domestication and found that strains in the Wine/European and Pacific West Coast Wine clades have gene copy number variation reflective of adaptations to the wine-making environment. The “wine circle/Region B”, a cluster of 5 genes acquired by horizontal gene transfer into the genome of commercial wine strains is also present in the majority of the British Columbian strains in the Wine/European clade but in a minority of the Pacific West Coast Wine clade strains. Previous studies have shown that *S. cerevisiae* strains isolated from Mediterranean Oak trees may be the living ancestors of European wine yeast strains. This study is the first to isolate *S. cerevisiae* strains with genetic similarity to nonvineyard North American Oak strains from spontaneous wine fermentations.

## Introduction

Wine is the product of alcoholic fermentation of grape juice typically by the yeast *Saccharomyces cerevisiae* (*S. cerevisiae*). The majority of the flavor and aroma-related compounds in wine are produced during alcoholic fermentation by *S. cerevisiae* (Fleet 2003). Wine can be produced by either of the following methods: inoculated fermentation, when a commercially prepared, single strain *S. cerevisiae* inoculum is used; or spontaneous fermentation when yeast inhabitant on the grapes from the vineyard or winery equipment carries out the fermentative process. In commercial wine production, a monoculture of domestic *S. cerevisiae* is typically inoculated into grape juice to ensure consistency and predictability during wine fermentation (Heard and Fleet 1985). Commercial wine strains, however, lack genetic diversity, which in turn leads to phenotypic redundancy, less complexity in wine flavor, and, therefore, lower value wine (Borneman *et al*. 2016). An important factor in wine flavor, aroma, and overall marketability is *terroir*, a term used to describe the effect of the complete natural environment in which grapes and wine are produced (van Leeuwen and Seguin 2006; Gilbert *et al*. 2014; Bagheri *et al*. 2017). The majority of the ∼250 commercial wine strains available on the global market have been isolated from vineyards or fermentations in Europe and may not be the ideal strains to reflect the *terroir* of non-European vineyards (Pretorius 2020). In increasingly competitive wine markets, small regional wineries such as those in the Okanagan Valley of British Columbia (BC), Canada must differentiate their products from their international competitors by making high-value wines that express the unique character, or *terroir*, of their region. The Okanagan Valley is the major wine region of BC, Canada that contains 84% of the province's vineyard acreage. The Okanagan Valley spans from the United States of American (USA) border with Washington State to approximately 250 km north and has a climate range from warm to cool and arid (Senese *et al*. 2012).

Differentiation among microbial communities from different wine regions and even closely situated vineyards has been demonstrated for fungal consortia (Bokulich *et al*. 2014; Morrison-Whittle and Goddard 2018; Knight *et al*. 2020; Liu *et al*. 2020; Martiniuk *et al*. 2023). As well, genetic differentiation among *S. cerevisiae* communities in vineyards and wineries has been observed (Knight and Goddard 2015; Cheng *et al*. 2020; Liu *et al*. 2020). Notably, region-specific *S. cerevisiae* populations produce wine with different chemical profiles suggesting that there is a microbial aspect to *terroir* (Knight *et al*. 2015). To make wine that reflects the local *terroir* from both a grape and microbial perspective, wineries may choose to carry out spontaneous fermentation, which is the traditional method of wine fermentation. Spontaneous fermentation is characterized by a diverse succession of yeast species present in the vineyard and winery and can produce more organoleptically complex wines with greater regional character, thus increasing the wine's market value (Fleet 2008; Knight *et al*. 2015). However, spontaneous fermentations are risky because *S. cerevisiae* may be in low abundance or spoilage yeast may be present, resulting in stuck or sluggish fermentations and the production of off-flavours (Fleet 2003). One approach to mitigate the risk of a stuck fermentation but create a wine that reflects the local *terroir* is to isolate indigenous strains from winery spontaneous fermentations and propagate the strains for use as starter cultures (Lopes *et al*. 2007; Schvarczova *et al*. 2017; Agarbati *et al*. 2018; Capece *et al*. 2019). This approach also requires that the strain first be genotyped using microsatellite markers and/or have its genome sequenced to demonstrate that the strain is not a commercial strain that the winery may, or may not, have used in previous fermentations (Legras *et al*. 2005; Richards *et al*. 2009; Hall *et al*. 2011; Martiniuk *et al*. 2016; Scholl *et al*. 2016).

*S. cerevisiae* wild lineages are mostly isolated from tree habitats (typically of the family *Fagaceae*), such as tree bark, decomposing wood, or the surrounding soil (Almeida *et al*. 2015; Pontes *et al*. 2020; Bai *et al*. 2022; Lee *et al*. 2022). Extensive sampling from primeval forests strongly supports that China and Taiwan are the origins of the *S. cerevisiae* species (Wang *et al*. 2012; Duan *et al*. 2018; Peter *et al*. 2018; Lee *et al*. 2022). The domestication of wine yeast strains is estimated to have occurred ∼7 to 12,000 years ago (Fay and Benavides 2005; Almeida *et al*. 2015; Legras *et al*. 2018). Early *S. cerevisiae* population genomic studies found that *S. cerevisiae* strains isolated from different geographical wine regions are often closely related, likely due to human movement of the strains, and suggestive of a single domestication event (Liti *et al*. 2009; Schacherer *et al*. 2009). As more *S. cerevisiae* strains have been sequenced from global wine regions, the data further confirm that the isolated strains are genetically very similar and have undergone population expansion after a domestication bottleneck, called the Wine/European (WE) clade (Almeida *et al*. 2015; Strope *et al*. 2015; Borneman *et al*. 2016; Gallone *et al*. 2016; Goncalves *et al*. 2016; Legras *et al*. 2018; Peter *et al*. 2018; Basile *et al*. 2021). Despite being isolated from around the globe, the WE clade strains share common ancestry with European commercial wine strains, suggesting the dispersal of European strains to non-European regions. For example, a sequencing study of *S. cerevisiae* strains isolated from New Zealand wine regions suggests population expansion following the adaptation of the WE clade strains to New Zealand (Higgins *et al*. 2021). Evidence suggests that the *S. cerevisiae* WE population is domesticated from wild Mediterranean oak tree strains as the nucleotide divergence between strains in the WE clade and Mediterranean oak clade is significantly lower than between strains in the WE clade and strains in the North American or Japanese oak clades (Almeida *et al*. 2015).

Domestication in the context of yeast and other microorganisms used in food and beverage production refers to the artificial selection and cultivation of wild populations for specific purposes, such as the production of bread, beer, and wine (Steensels *et al*. 2019). The genomic signatures of domestication in *S. cerevisiae* strains include gene copy number variation (CNV), horizontal gene transfer (HGT), single nucleotide polymorphisms (SNPs), heterozygosity, and genome decay (Steensels *et al*. 2019; Bai *et al*. 2022). For example, the commercial wine strain EC1118 contains 3 genome regions acquired by HGT, designated as A, B, and C (Novo *et al*. 2009). Region B, also known as the wine circle, is a 17 kb segment acquired from *Zygosaccharomyces bailli* (*Z. bailli*) that encodes a putative oxoprolinase, a nicotinamide transporter, Flo11, and 2 transcription factors with similarity to Put3 and Upc2 (Borneman *et al*. 2011; Galeote *et al*. 2011; Legras *et al*. 2018). Despite its name, Region B/wine circle has also been identified in cocoa, bread, beer, bioethanol, and olive strains (Pontes *et al*. 2020). With increased sequencing output of *S. cerevisiae* strains from a variety of niches, a total of 42 new genome regions have been identified that are not present in the S288c lab strain, including 12 regions of HGT from non-*Saccharomyces* species (Legras *et al*. 2018). These non-S288c genes are part of the *S. cerevisiae* pan-genome that contains 7,796 open reading frames (ORFs) as compared to the S288c lab strain genome that contains 6,081 nonredundant ORFs (Duan *et al*. 2018; Peter *et al*. 2018). The *S. cerevisiae* pan-genome is a mixture of ancestral genes, genes acquired by hybridization with other *Saccharomyces* species (introgression) or HGT as mentioned above (Borneman *et al*. 2016; Duan *et al*. 2018; Legras *et al*. 2018; Peter *et al*. 2018). Importantly, the non-S288c genes present in the genomes of industrial strains provide an advantage in a particular niche such as the *FOT* genes that encode oligopeptide transporters (Region C) that allow efficient utilization of nitrogen resources during wine fermentation (Marsit *et al*. 2015, 2016).

The majority of *S. cerevisiae* strains isolated from wine regions with whole genome sequencing (WGS) data are from European countries, with Australian, New Zealand, South African, and South American strains included as well (Almeida *et al*. 2015; Strope *et al*. 2015; Borneman *et al*. 2016; Legras *et al*. 2018; Peter *et al*. 2018; Basile *et al*. 2021; Higgins *et al*. 2021). The few *S. cerevisiae* strains isolated from US wine regions that have been sequenced fall into the WE clade and no *S. cerevisiae* yeast isolated from Canadian wine regions have been sequenced, even though Canada is home to multiple wine regions (Peter *et al*. 2018). In this study, we carry out the first whole genome sequencing of 75 *S. cerevisiae* strains isolated from spontaneous fermentations of grapes sourced from the Okanagan Valley wine region in BC, Canada, referred to as the “BC” strains. While the majority (38) of the 75 BC strains fall into the WE clade, 34 belong to a new clade that we have named the Pacific West Coast Wine (PWCW) clade. We provide evidence that the PWCW clade is descended from North American oak tree strains and is partially domesticated due to adaptation to a wine fermentation environment. Our data suggest that wine regions can harbor genetically distinct strains and should be further explored as a source of *S. cerevisiae* strain diversity.

## Materials and methods

### *S. cerevisiae* strain isolation, sequencing, and quality filtering

The 75 *S. cerevisiae* strains used for this sequencing project were isolated from early-, mid-, and late-stage spontaneous fermentations carried out with grapes from 3 different regions of the Okanagan Valley (Supplementary Table 1). Spontaneous fermentations were either performed in a winery, or grapes were picked from a vineyard and brought back to the lab for crushing and fermentation (Cheng *et al*. 2020; Martiniuk *et al*. 2023). Microsatellite data analysis on a collection of ∼250 genotypes of BC *S. cerevisiae* strains isolated from wineries and vineyards in the Okanagan Valley was used to identify 75 strains for WGS that had different genotypes (Supplementary Table 2) (Cheng *et al*. 2020; Martiniuk *et al*. 2023). DNA was extracted using phenol/chloroform and sent to the Michael Smith Genome Sciences Centre where sequencing libraries were prepared with Genome Shotgun PCRFree 1.3 and Index TruSeq paired-end. One hundred and fifty base pair, paired-end libraries were sequenced on a HiSeqX-1. An average of 15 million reads was generated per genome resulting in 205-fold average genome coverage.

### Variant calling and phylogenetic tree construction

Raw reads were mapped to the *S. cerevisiae* S288c R64-3-1 reference genome assembly using bwa-mem2 (v 2.0) (Engel *et al*. 2014; Vasimuddin *et al*. 2019). Optical duplicates were tagged using samblaster (v 0.1.24) (Faust and Hall 2014) and mapped reads were filtered using samtools (v1.14) flag (-F 2316) to exclude reads that were unmapped, mate unmapped, not primary alignment, and supplementary alignment (Li *et al*. 2009). The mapped reads were further filtered to a quality score of q10 using samtools for use in variant calling. Unfiltered reads were used for subsequent CNV calling. Variants were called using Deepvariant (v1.4.0) with the WGS model type (Poplin *et al*. 2018). Joint genotype calling was performed on the resulting gvcfs using GLnexus (1.4.1) with default parameters and DeepVariant WGS configuration (Yun *et al*. 2021). After joint genotyping, the gvcf contained 1,739,284 variants. To analyze phylogeny, the joint called gvcf was filtered based on missingness, minor allele count, and quality using VCFtools (v0.1.15) with the parameters (–max-missing 1 –remove-indels –mac 3 –minQ 10) resulting in 477,158 kept variant sites (Danecek *et al*. 2011). The resulting gvcf was converted to phylip format using vcf2phylip (v2.8) (Ortiz 2019). The phylip was then used as input to IQtree(v 2.2.0) with parameters (-m MFP -alrt 1000 -bb 1000) using CBS432 as an outgroup to root the tree (Kalyaanamoorthy *et al*. 2017; Hoang *et al*. 2018; Ortiz 2019; Minh *et al*. 2020). The best substitution model selected was TVMe+R3, which was used by IQtree for the maximum-likelihood (ML) phylogenetic tree generation. The resulting ML phylogenetic tree was then visualized using ITOL v5 including bootstrap values (Letunic and Bork 2021).

### Population admixture and statistics

The filtered gvcf was pruned for linkage disequilibrium using plink parameters (–indep-pairwise 50 5 0.5) (Purcell *et al*. 2007). ADMIXTURE (v 1.3.0) was run on the pruned bed file for K 1–60 (ancestral populations) with cross-validation set to 10 (Alexander *et al*. 2009). A K value of 34 was selected with a low cross-validation error. The output of Admixture K 34, Q values were then plotted as a bar plot in ITOL on the phylogenetic tree. Strains were assigned to clades based on the phylogenetic tree, and the tree was collapsed to the condensed clades for downstream analysis excluding the “Other” clade as it only had a single isolate. Dsuite (v0.5 r44) trios were run on each trio of clades using default parameters using the same pruned LD gvcf as input for the phylogenetic tree, and the collapsed tree as input, generating D statistics, Z-score, unadjusted *P*-values, the f4-ratios, and counts of the BBAA, BABA, and ABBA patterns (Malinsky *et al*. 2021). Dsuite f-branch was used to produce and plot an f-branch matrix using the previously generated f4-ratios and the condensed tree to estimate the presence of gene-flow between populations (Malinsky *et al*. 2018). Genome-wide Fst pairwise comparisons were made using VCFtools (v0.1.17) with the parameters (–weir-fst-pop –fst-window-size 10,000) on the original gvcf containing 1,739,284 variants (Danecek *et al*. 2011). Nucleotide diversity and Tajima's D value were calculated similarly using VCFtools.

### Heterozygosity

The degree of heterozygosity for each strain was analyzed by examining the proportion of heterozygous variants amongst all variants present across the entire genome using an in-house script. This proportion was calculated by applying a 50 kb sliding-glass window (with 25 kb steps) to each genome and determining the number of heterozygous variants relative to the total number of variants within each window (Borneman *et al*. 2016). The distribution of these ratios across the entire genome was plotted to examine the variation in heterozygosity present between different genomic sites. The total percent heterozygosity for each strain was defined as the median of all ratios. Heterozygous sites with allelic balances below 0.3 were filtered out using gatk (v4.2.0.0) (Van der Auwera and O’Connor 2020).

### Annotation and analysis of variants

The original gvcf was filtered at a minimum depth of 10 and quality of 10 using VCFtools (v0.1.17) to identify high-quality SNPs andinsertion–deletions, and split by sample. Filtered VCF files were analyzed via EnsemblFungi (https://fungi.ensembl.org/index.html) Variant Effect Predictor (VEP) for variant annotation using S288c as a reference genome (McLaren *et al*. 2016). High-impact variants were identified and defined as causing probable loss of function (LOF) within coding regions via frameshift mutation, loss of start codon, or premature stop codon within 98% of the coding region (Bergstrom *et al*. 2014). As VEP does not analyze variants holistically, the effect of one high-impact variant on another variant is not considered. To remedy this, frameshift variants within the same coding region that retained an in-frame reading were removed from the analysis using an in-house script; however, LOF has not been experimentally validated and is solely a prediction. SNPs predicted to be deleterious to protein structure and function were identified using SIFT4G (Vaser *et al*. 2016). Gene ontology (GO) enrichment was performed using YeastMine (https://yeastmine.yeastgenome.org/yeastmine/begin.do) with a Benjamini–Hochberg test correction (Hochberg and Benjamini 1995). K-means clustering was conducted to group LOF genes together based on strains with shared high-impact variants. The number of K was chosen using the gap statistic (Tibshirani *et al*. 2002).

### Gene copy number variation

Using the GFF files, the number of reads mapped to each open reading frame (ORF) was normalized to the length of the gene to assess coverage and completeness. Bam files of previously unfiltered mapped reads were then analyzed using CNVkit (Talevich *et al*. 2016). The log2 ratios for segments of around 120 bp were performed in downstream steps of CNVkit using the HMM-based method. The base coverage was calculated from the median coverage along the genome. The log2 ratio was calculated by taking the base coverage and comparing it to the query segments’ coverage generating a ratio, and then taking the log2 of that ratio. For example, if the median coverage for the sample is 100, and a segment has 200× coverage, the log2 ratio would be log2(200/100), resulting in a log2 ratio of 1. CNV kit call was used to estimate absolute CNV from these ratios using the parameter “threshold” and ploidy was adjusted accordingly (Talevich *et al*. 2016).

### Differential gene copy number variation

The mean absolute copy number (CN) values for ORFs were used for differential CNV analysis. Only diploid strains listed in Supplementary Table 4 were considered for the analysis, which disqualified the haploid strain YPS163_1b. CN values were filtered for ORF loci within the top 50th percentile of variance. CN values of 0.7 or less were considered absent (zero), otherwise, values were rounded to the closest integer. Differential ORF CN loci were determined with a Kruskal–Wallis test, with a *P*-value cutoff and false discovery rate (FDR) of 0.05. To identify genes with a potential for strong phenotypic impact, genes were included in the analysis if they had a mean CN value >2.5 or <1 for at least 1 of the strain groups.

### Gene loss

Mean ORF CN was used to analyze gene loss in the 4 diploid strain groups described in Supplementary Table 4. Gene CN was rounded in the same way as for differential CN analysis; however, CN values >1.5 were all rounded to 2. Therefore, CN values for ORFs could be 0, 1, or 2. Total gene loss was calculated by taking strain ORF CN sums subtracted from the total possible gene CN (14172). For homozygous gene loss, presence-absence was determined by rounding all ORF CN >1 to 1 and then subtracting the total number of measured ORFs (7,086) by the sum of the ORFs present in each strain. Finally, heterozygous CN was determined by subtracting the total gene loss from the homozygous gene loss events for each strain. A Wilcoxon Rank Sum test was employed to determine the statistical significance of gene loss between strain origins.

### Chromosome ploidy

The ploidy of the 75 BC wine yeast strains sequenced in this study was determined using methods previously described (Peter *et al*. 2018). Briefly, yeast strains were grown to log phase and fixed in 70% ethanol overnight at 4°C. Cells were spun down and resuspended in 15 uM propidium iodide and analyzed using a CytoFlex flow cytometer with CytExpert v2.4 software. Cell density was plotted against fluorescence intensity at 610 nm to determine the distribution of G1/G2 peaks for each strain, and the ploidy was subsequently determined by comparing the mean fluorescence intensity for each isolated strain to *S. cerevisiae* strains of known ploidy. Ploidy was further confirmed by analyzing the ratio of read depth for reference and alternative alleles at heterozygous sites. The expected ratio of read depth at heterozygous sites is ½ for diploid strains and ⅓ or ⅔ for triploid strains. Aneuploidy was confirmed using coverage differences between chromosomes. Reads mapped to the S288c genome were analyzed using a CNV-kit batch with a 10,000 bp target size and in WGS mode (Talevich *et al*. 2016). Aneuploidy was predicted from output figures showing increasing or decreasing coverage along entire chromosomal regions. Raw reads for the S288c reference genome (accession SRR2155774) were downloaded and run through the pipeline for comparison. The resulting bam file was compared to the previously mapped reads using the genome browser IGV to compare raw coverage and consistency with CNVkits output (Thorvaldsdottir *et al*. 2013; Talevich *et al*. 2016).

### Non-S288c gene identification and manual filtering

All reads from the 75 BC strains that did not map to S288c were assembled with Abyss using every second kmer length between 60 and 150 (Jackman *et al*. 2017). For the 34 global strains, we downloaded assemblies from the NCBI where possible and assembled reads with Abyss using every second kmer length between 60 and 150 when necessary. The assemblies with the largest N50 were selected and ORFs were predicted using Augustus v2.5.5 and trained on S288c (Stanke *et al*. 2006). ORFs > 180 bps (60 amino acids) were then clustered using cd-hit in order to reduce the number of ORFs and identify ORFs that are the same among samples (Fu *et al*. 2012). Each cluster of ORFs contained nucleotide sequences 97% or more identical to one another, adjusting for sequence length. A total of 1,432 clusters were identified. Clusters that contained ORFs present in the global strains but not the 75 BC strains were discarded, leaving 259 clusters. Any sequence that did not contain both a start and stop codon was discarded. The representative sequence from each cluster (the longest sequence in each cluster of ORFs 97% or more identical to one another) was cross-checked to ensure that at least 1 BC strain also contained the longest sequence, reducing the cluster number to 105. The representative sequence from each cluster was then tblastx-ed against 2 pangenomes and the RefSeq database (release v.201) for annotation and comparison (Supplementary Table 11). Multiple sequence alignment was performed using Clustal Omega, and the alignment was visualized using MView (Brown *et al*. 1998; Sievers *et al*. 2011).

### Heat maps for non-S288c genes

Heatmaps were generated for selected genes using the pheatmap package in R. Selected genes were visualized for presence (full and partial length)/absence in the 75 BC strains. The nucleotide sequences of 105 non-S288c clusters were blasted (blastn, bit score ratio of 0.4 and E-value <0.000001) against global strains of interest for which assemblies exist or for which reads of adequate quality and coverage were available for assembly. To assemble these, we used MEGAHIT v1.2.9 with parameters “–no-mercy –prune-level 3 –min-count 5” (Li *et al*. 2015, 2016). The contigs were then corrected and scaffolded using the S288c reference genome R64-3-1 with ragtag v 2.01 (Alonge *et al*. 2019). Contigs less than 25,000 bp were removed from the assembly leaving 17 scaffolds, comprising the 16 nuclear chromosomes and 1 mitochondrial genome. Fastq files were not available for all 34 global strains; therefore, the data quality, assembly methods, and coverage differed for all strains, making these data less consistent than our 75 BC strains (Minoche *et al*. 2011; Chen *et al*. 2013; Lou and Therkildsen 2021). Because we removed clusters that did not contain sequences from our 75 strains, and because the sequencing quality, type, and depth were inconsistent as these samples are from the public domain, we visualized the non-S288c cluster in our 75 BC strains independent of the 34 global strains.

## Results

### A genetically diverse collection of *S. cerevisiae* strains from BC, Canada

**Fig. 1.**
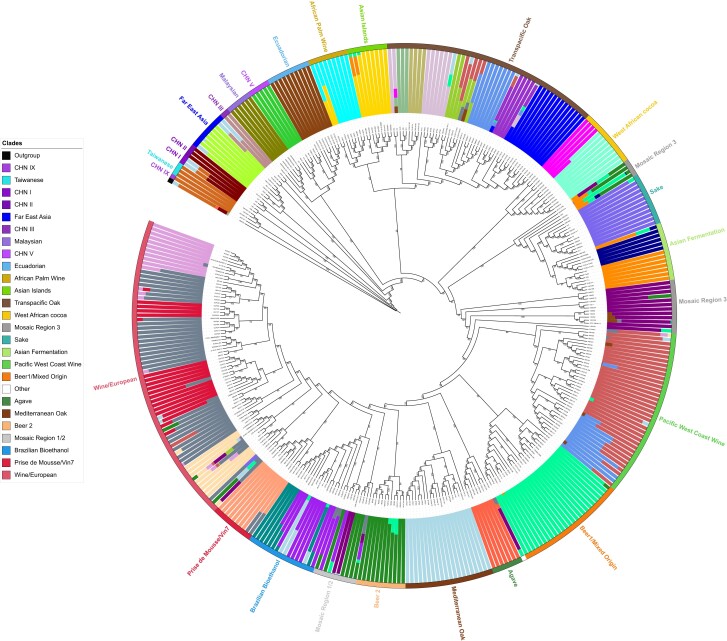
Maximum likelihood phylogenetic tree and associated admixture of 75 BC and 296 global *S. cerevisiae* strains. The tree was constructed using 477,158 genome-wide SNPs with *S. paradoxus* isolate CBS432 used as an outgroup. Ultra-fast bootstrap values are indicated on the branches and the legend indicates the color and labels corresponding to each clade as defined by the tree and the outer circle. Population structure using ADMIXTURE analysis at *K* = 34 was selected, based on the lowest cross-validation error score, and is presented on the inner circle.

From 2013 to 2018, we isolated *S. cerevisiae* strains from spontaneous wine grape fermentations carried out with both pinot gris and pinot noir grapes from the Okanagan Valley in BC. Grapes were either taken directly from a vineyard to the lab and spontaneously fermented or fermented in the winery and sampled from winery fermentations. Depending on the study, we isolated *S. cerevisiae* from early-, mid-, and late-stages of fermentation (Supplementary Table 1). To differentiate commercial wine yeast strains from noncommercial strains we used microsatellite analyses based on 10 repetitive loci (Martiniuk *et al*. 2016; Cheng *et al*. 2020). Based on our microsatellite results, we chose 75 BC *S. cerevisiae* strains for WGS using the Illumina Hi-Seq platform (Supplementary Fig. 1, Supplementary Table 2). We obtained 150 bp paired-end reads with an average of 15 million reads per genome and 205-fold genome coverage. An average of 97% of sequencing reads mapped back to the S288c *S. cerevisiae* reference genome with an average density of 62,611 SNPs. A previous study that sequenced 1,011 *S. cerevisiae* genomes identified 26 clades for the *S. cerevisiae* species, which often diverged based on geographic origin, ecological niche, and industrial application (Peter *et al*. 2018). To compare BC strains with global populations, we mapped reads from 75 BC strains and 296 previously sequenced global strains (a total of 371 strains) to the *S. cerevisiae* S288c reference genome. Global strains were selected to represent a variety of wild and domesticated lineages including 10 commercial wine strains (Supplementary Table 3). A maximum-likelihood phylogenetic tree was constructed using biallelic SNP data that revealed 25 clades, consistent with previous *S. cerevisiae* population genomic studies (Fig. 1) (Peter *et al*. 2018; Pontes *et al*. 2020; Han *et al*. 2021). The clades were named based on the global representative strains with the exception of a new clade that we have named the Pacific West Coast Wine (PWCW) clade and a clade that we have named the Transpacific Oak (TPO) clade (Fig. 1). These strain lineages are congruent with previous evidence of an out-of-China origin for the species *S. cerevisiae* (Wang *et al*. 2012; Duan *et al*. 2018; Peter *et al*. 2018).

Our analysis finds the WE clade as the most populated clade (80/371 total strains) including the majority of the BC strains (38/75). The WE clade contains commercial wine, vineyard, bakery, cider, and human clinical strains. A previous sequencing study of 212 wine yeast strains, including 106 commercial wine strains, found that most strains clustered in a highly related wine clade with little genetic variation (Borneman *et al*. 2016). Adjacent to the WE clade is the *Prise de Mousse*/Vin7 clade that contains strains previously described as 2 lineages—*Prise de Mousse* (strains used for champagne production) and Vin7 (fructophilic strains) (Borneman *et al*. 2016; Pontes *et al*. 2020). A recent sequencing study of 65 oenological strains from an Italian Biotechnology company that included analysis of 503 strains from the Borneman *et al* (2016) and Peter *et al* (2018) studies, corroborated these findings (Borneman *et al*. 2016; Peter *et al*. 2018; Basile *et al*. 2021). Our phylogenetic tree resolved 10 *Prise de Mousse*/Vin7 strains including the BC strain SBV155 (Fig. 1). Evidence suggests that the closest wild relative of commercial wine yeast strains are *S. cerevisiae* strains isolated from oak trees in the Mediterranean (Almeida *et al*. 2015). We included 20 Mediterranean oak tree strains in our phylogenetic tree and show that they indeed cluster near the WE clade that contains commercial wine strains (Fig. 1).

Interestingly, we find that 34 of the 75 BC strains fall into a new clade that we have named the PWCW clade. In addition to the 34 strains from this study, 1 additional strain previously isolated from BC (under 2 unique sample aliases MTF2421 and ZP611), and 2 strains isolated from California, USA (UCD 05-780 and YM1527) belong to the PWCW clade. The PWCW clade has a higher relative sequence diversity when compared to the WE clade (π = 3.5 × 10^−3^ vs 1.8 × 10^−3^, respectively) (Fig. 1, Supplementary Fig. 2). BC strains in the PWCW clade was isolated from all stages of fermentation, with the majority (31/34) isolated from the late stage (65–90% sugar depletion) spontaneous fermentations (Supplementary Table 1). We did not find any correlation between the fermentation stage or the origin (vineyard vs winery) that the strain was isolated from when comparing strains in the WE and PWCW clades (Supplementary Table 1). In many cases, the same strain was isolated from both vineyard and winery fermentation (Supplementary Table 1). Unexpectedly, we also find that 1 of our BC strains (OK047, isolated from a vineyard grape spontaneous fermentation) clusters in the TPO clade (Fig. 1). The OK047 strain was isolated from a late-stage wine fermentation with more than 10 isolates, suggesting that it is not an environmental contaminant but can actively ferment. The TPO clade contains strains isolated mostly from oak trees or otherwise arboreal-related habitats and combines lineages from several different countries including China, far-east Russia, Japan, and the USA as a unified clade that is similar to a previously described “Clade 17” (Fig. 1) (Pontes *et al*. 2020). Further, our results support the present theory that an expansion of Asian lineages leads to the colonization of wild oak strains in North America (Duan *et al*. 2018; Peter *et al*. 2018; Pontes *et al*. 2020). We were also surprised to find that 2 BC strains, P93A01 and P93F02, cluster with strains from the Beer 1/Mixed Origin clade, a clade that was first described by previous sequencing projects focused on *S. cerevisiae* beer strains (Gallone *et al*. 2016; Goncalves *et al*. 2016).

The population structure of the different phylogenetic clades was analyzed using ADMIXTURE at *K* = 34 (Fig. 1). In the TPO clade, as previously seen, oak tree strains isolated from Pennsylvania, USA (dark blue) can be resolved from oak tree strains isolated from North Carolina, USA (light blue) (Almeida *et al*. 2015; Tilakaratna and Bensasson 2017). Based on our admixture data, we also find that the BC strain OK047 shares a population structure with the North Carolina oak strains (Fig. 1). To our knowledge, this is the first time that a strain isolated from a spontaneous wine fermentation has clustered with North American oak strains. Our admixture data of PWCW strains also indicates that the clade forms 2 subpopulations that show similarity to TPO strains, specifically North Carolina Oak (light blue) and a cluster of mosaic strains (rust). Notably, there is minimal admixture between WE and PWCW strains, apart from 2 PWCW strains (P104A01 and SBV087) and one WE strain (SBV171). We also observe some admixture of wild Ecuadorian strains within PWCW (brown). This data suggests that the closest wild relative of PWCW strains are *S. cerevisiae* strains isolated from oak trees in North America.

**Fig. 2.**
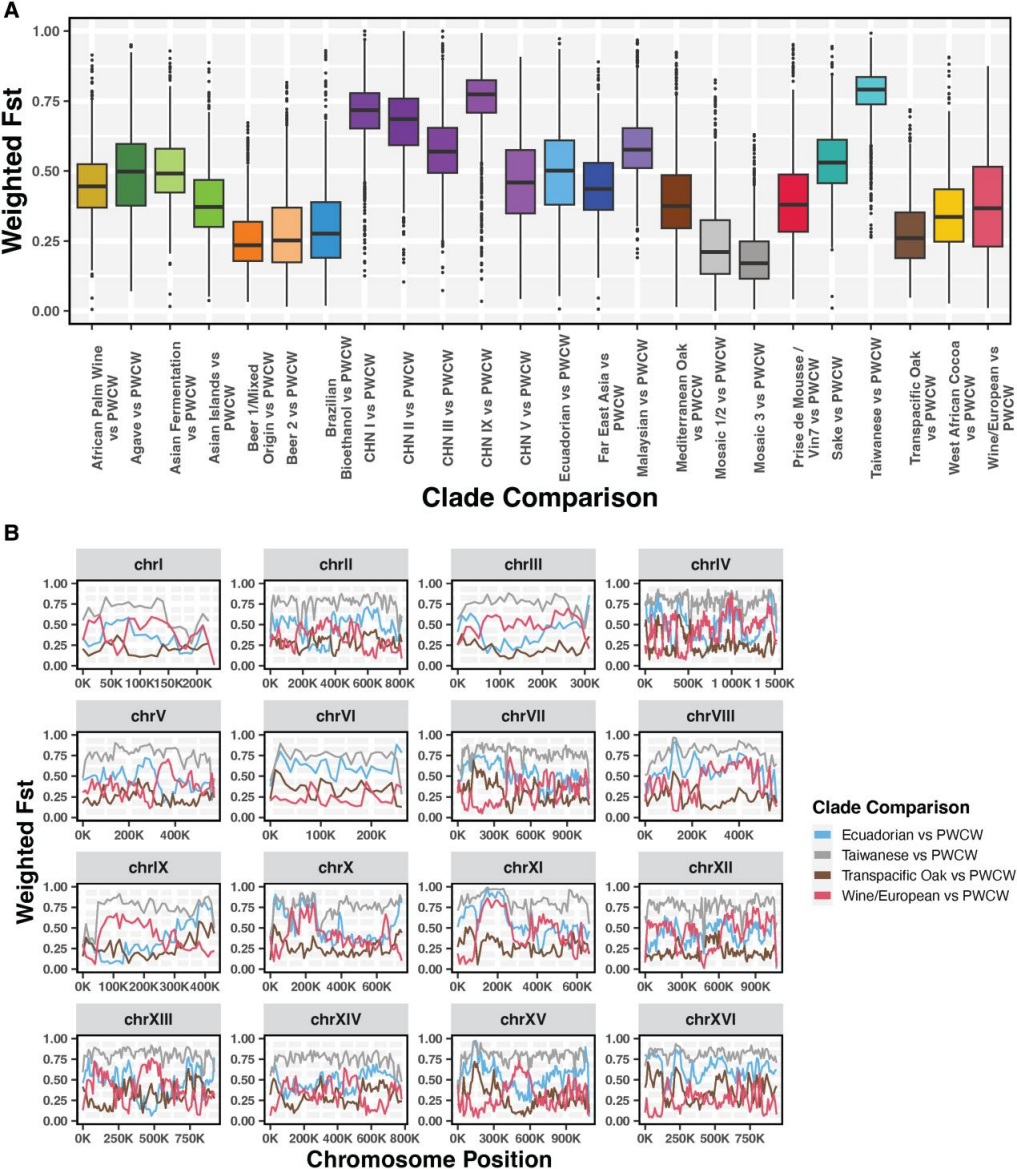
Genome-wide pairwise Fst comparisons show gene flow between PWCW and WE, TPO, and Ecuadorian clade strains a) Pairwise Fst calculated using a 10 kb sliding-windows approach between PWCW and 24 phylogenetic clades. Boxplots show the distribution of weighted Fst comparisons, with the black line indicating the median. Colors correspond to clade groups in Fig. 1. b) Genome scan of weighted Fst (10 kb sliding-windows) between PWCW and WE, TPO, Ecuadorian, and Taiwanese clade strains, split by chromosome.

Microbial range expansion of WE strains has previously been observed within New Zealand vineyards, likely a result of human-associated introduction (Higgins *et al*. 2021). The phylogenetic position of the New Zealand strains in our tree is within the WE clade (Fig. 1). In contrast, the branching of the PWCW clade in the tree precedes that of the WE clade, suggesting that the PWCW population did not expand from the WE clade strains into the Okanagan Valley (Fig. 1, Supplementary Fig. 2). Furthermore, the evolutionary divergence between PWCW and WE clade strains occurred more recently than that of WE and TPO clade strains, suggesting that PWCW clade strains may be intermediary to TPO and WE clade strains. To further examine the evolutionary origins of the PWCW strains, we conducted a genome-wide scan of population differentiation using the Weir and Cockerham Fst estimator (Weir and Cockerham 1984). Pairwise Fst was calculated in 10 kb windows between PWCW and the other 24 clades, and the mean was plotted (Fig. 2a). The PWCW population demonstrated similarity, based on low median Fst values, to the TPO clade as well as clades with European origins (Beer 1/Mixed origin, Beer 2, Brazilian Bioethanol, WE, Mediterranean Oak), and Mosaic clades. Evidence of allele sharing was further supported by analyzing pairwise f-branch statistics between clades, demonstrating the contribution of both TPO and WE lineages to PWCW (Supplementary Fig. 3). The f-branch statistic between Ecuadorian and PWCW clades also suggests gene flow between these populations (Supplementary Fig. 3). Indeed, an examination of gene flow along the individual chromosomes of the PWCW population demonstrated regions with similarity to TPO, WE, and Ecuadorian *S. cerevisiae* genomes (Fig. 2b). To validate our analysis, we calculated pairwise Fst between the PWCW and Taiwanese clade strains and observed no evidence of gene flow, as expected (Fig. 2b). Overall, this analysis of phylogeny and population structure indicates PWCW strains contain genomic contributions from both wild (TPO, Ecuadorian) and domestic (WE) clades.

### BC strains in the WE, PWCW, and TPO clades are diploid whereas BC strains in the Beer 1/Mixed Origin clade are triploid

**Fig. 3.**
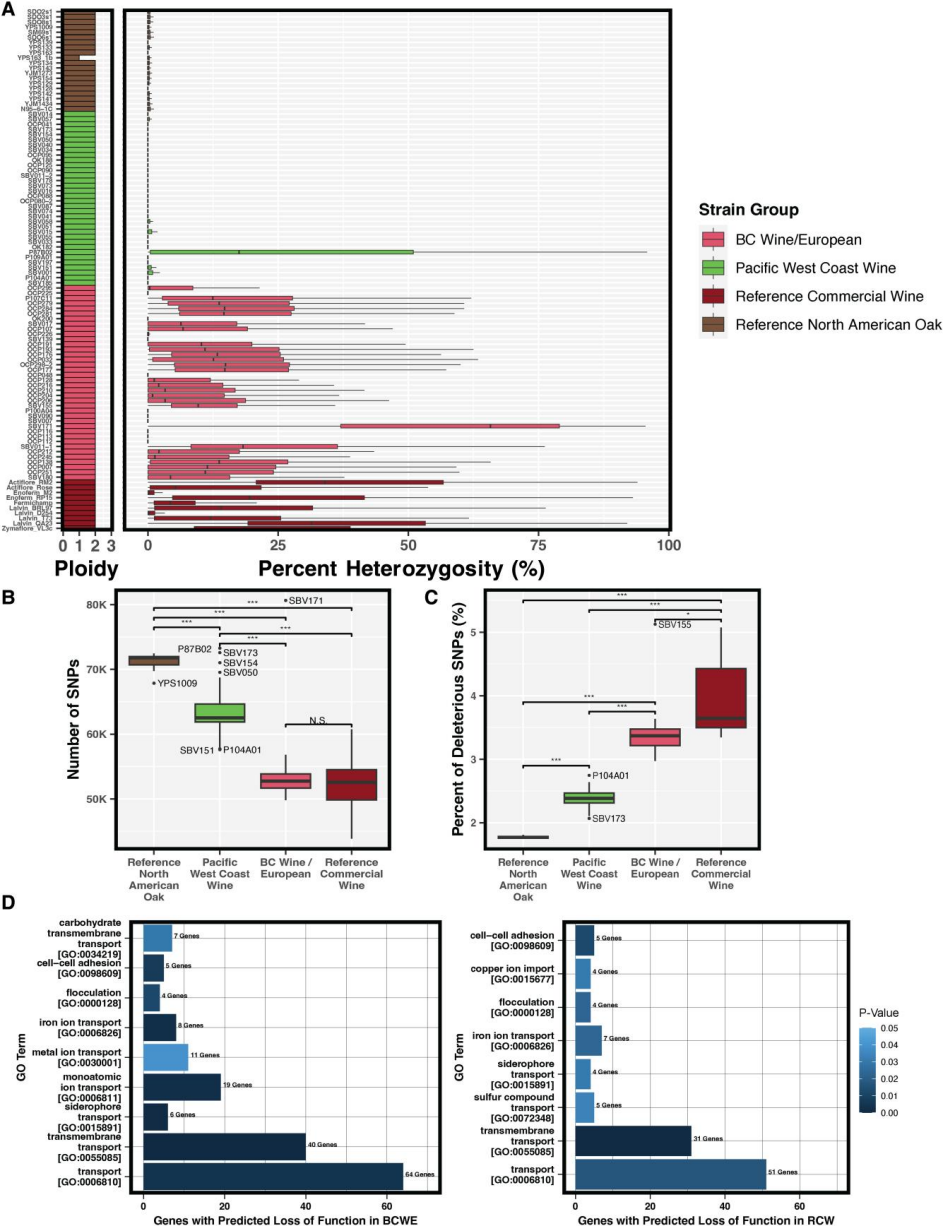
Genomic signatures of PWCW strains suggest partial domestication. a) Ploidy and percent heterozygosity of 38 BCWE, 34 PWCW, 20 RNAO, and 10 RCW *S. cerevisiae* strains. Boxplots show the distribution of heterozygosity in 50 kb regions across the genome, with the black line indicating the median, b) Total number of SNPs across all strains for each strain group, and c) Proportion of coding SNPs predicted to be deleterious for each strain group. Boxplots show the distribution in number within strain groups, with the black horizontal line indicating the median. Outlier strains are indicated above and below the boxplots. For (b) and (c), N.S. indicates no significant difference, *P = 4.8 × 10^−6^, ***P < 2 × 10^−16^. d) GO biological process enrichment analysis of S288c genes that contain a high-impact variant within at least 1 strain of the BCWE and RCW strain groups.

The ploidy of the 75 BC *S. cerevisiae* strains was determined by analyzing their relative cell DNA content via flow cytometry. Wine yeasts are typically diploid with limited occurrences of aneuploidy whereas beer yeasts exhibit both aneuploidy and increased ploidy (Gallone *et al*. 2016; Duan *et al*. 2018; Peter *et al*. 2018; Scopel *et al*. 2021). Consistent with these expectations, the majority of BC strains (73/75) were diploid, including all BC strains within the WE, PWCW, and TPO clades (Fig. 3a). The remaining 2 BC strains, P93A01, and P93F02, that fall within the Beer 1/Mixed Origin clade were both observed to be triploid. To determine the aneuploidy of specific chromosomes, we used CNVkit (Talevich *et al*. 2016). We observed that SBV180, a diploid BC strain in the WE clade has one extra copy of chromosome XII which is one of the larger chromosomes in *S. cerevisiae* and is not commonly aneuploid (Scopel *et al*. 2021). As well, the triploid Beer 1/Mixed Origin clade strains P93A01 and P93F02 both have an extra copy of chromosome IX, which happens to be the second most common aneuploidy identified in yeast strains (Peter *et al*. 2018; Scopel *et al*. 2021).

### Limited heterozygosity in PWCW clade strains

To examine the genomic traits of PWCW strains in more detail, we selected 102 strains that were placed into the following 4 groups: 1. BCWE—the 38 BC strains in the WE clade; 2. PWCW—the 34 BC strains in the PWCW clade; 3. RCW—a subset of 10 reference commercial wine strains from the WE clade; 4. RNAO—a subset of 20 reference North American oak strains from the TPO clade (Supplementary Table 4). Heterozygosity is a genomic signature of domestication within *S. cerevisiae*, reflecting the impaired abilities of some domesticated strains to sporulate and undergo the mating life cycle (Borneman *et al*. 2016; Gallone *et al*. 2016; Goncalves *et al*. 2016; Duan *et al*. 2018; Peter *et al*. 2018; De Chiara *et al*. 2022). Traits in wild strains such as high sporulation efficiency, inbreeding, and haplo-selfing reduce heterozygosity, and promote the removal of heterozygous deleterious mutations, a process described in Mortimer's genome renewal hypothesis (Mortimer *et al*. 1994; Muller and McCusker 2009; Magwene *et al*. 2011; Magwene 2014; Duan *et al*. 2018). The median heterozygosity and distribution of heterozygous regions across the genome were measured for all strains in the 4 groups (Fig. 3a). The BCWE strain group demonstrated a high degree of heterozygosity with 71% (27/38) of the strains containing heterozygous genomes and a median of 3.8% heterozygosity for the group. High heterozygosity was also observed in almost all RCW strains (median 13.0%). In contrast, all strains in the PWCW and RNAO groups, with the exception of P87B02 (PWCW clade), are homozygous and the median heterozygosity is 0% (Fig. 3a). The minimal heterozygosity of PWCW strains suggests that they have life cycle traits comparable to that of RNAO strains with regards to sporulation and haplo-selfing. SBV171, a BCWE strain, has the highest median heterozygosity of all strains analyzed at 65.8%. Interestingly, SBV171 also shows admixture between WE and PWCW clades, suggesting the high heterozygosity is a result of recent outcrossing (Fig. 1). To determine if our BC strains are homothallic or heterothallic, we analyzed the sequence of the *HO* endonuclease gene and found that all BC strains have a functional version of *HO*. Therefore, heterozygosity in BCWE strains may result from past outcrossing events and poor sporulation efficiency, both of which contribute to heterozygosity in *S. cerevisiae* and have been described in domesticated clades (Murphy and Zeyl 2010; Magwene *et al*. 2011; De Chiara *et al*. 2022).

### Increased SNP density but lower deleterious SNP accumulation in PWCW strains compared to RCW strains

Domesticated *S. cerevisiae* commercial wine strains lack genetic diversity and have reduced SNP density relative to wild strains when using S288c as the reference genome (Borneman *et al*. 2016; Duan *et al*. 2018; Peter *et al*. 2018). Due to lifestyle changes and selection pressure, domesticated strains also have a higher propensity for acquiring deleterious mutations (Warringer *et al*. 2011; Zorgo *et al*. 2012; Borneman *et al*. 2016; Gallone *et al*. 2016; Duan *et al*. 2018; Legras *et al*. 2018; Peter *et al*. 2018). The individual dissimilarity of strains within the BCWE group is the lowest of all 4 strain groups analyzed, as measured by an average nucleotide diversity (π) of 1.1 × 10^−3^ (Supplementary Fig. 2). Much of the genetic variation within the BCWE strain group is also found in low frequency, with a strongly negative Tajima's D value of −1.3, suggesting that the BCWE population has gone through a recent bottleneck event, as has been reported by others for commercial wine strains, which have a negative Tajima's D value of −0.4 in this study (Liti *et al*. 2009; Schacherer *et al*. 2009; Almeida *et al*. 2015; Borneman *et al*. 2016; Peter *et al*. 2018). In contrast, the PWCW strain group has higher dissimilarity and nucleotide diversity (3.5 × 10^−3^), and a positive Tajima's D value of 1.3 (Supplementary Fig. 2). These metrics indicate that PWCW strains are considerably more diverse than BCWE strains and that there is an absence of rare alleles in the PWCW population.

To compare SNP density between the 4 strain groups, the total number of SNPs relative to S288c was determined for each strain, and the median SNP density was calculated for each strain group (Fig. 3b). Amongst the 4 groups, RCW and BCWE strains have the lowest SNP density with median values of 52,544 and 52,738 SNPs, respectively, followed by PWCW with 62,508 SNPs, and RNAO with 71,783 SNPs. In the PWCW strain group, SBV050, SBV154, and SBV173 are upper outliers with 7,000–10,000 more SNPs than the median for PWCW strains, which supports their increased admixture with North American Oak strains (Fig. 1). Strain P87B02 is also an outlier with 10,760 more SNPs than the PWCW median, likely reflective of the high heterozygosity of this strain. In contrast, P104A01 is a lower outlier with 10,209 fewer SNPs than the median for PWCW strains, which is consistent with the strain's increased admixture with the WE clade (Fig. 1). The lower SNP density of BCWE and RCW strains compared with the higher SNP density of wild RNAO strains is consistent with observations of reduced SNP density in domesticated clades (Peter *et al*. 2018).

Next, deleterious SNPs were predicted using SIFT and their proportion relative to the total number of SNPs in protein-coding regions was calculated for each strain (Fig. 3c) (Vaser *et al*. 2016). Strains in the RCW group have the highest relative percentage of deleterious SNPs (median 3.6%), followed closely by BCWE (median 3.4%). In contrast, the PWCW and RNAO strain groups both have a significantly lower accumulation of deleterious SNP accumulations (median 2.4 and 1.8%, respectively, *P* < 2 × 10^−16^). Three RCW strains (Fermichamp, Actiflore RM2, and Lalvin QA23) and 1 BCWE strain (SBV155) in the *Prise de Mousse* lineage have a much greater percentage of deleterious mutations with 4.6–5.1% of all protein-coding SNPs predicted to be deleterious to protein structure and function. Strain P104A01 is also an upper outlier in the PWCW clade, which is again consistent with this strain showing increased admixture with WE clade strains (Figs. 1, 3c). This analysis indicates that PWCW strains are intermediaries to RNAO and BCWE/RCW strains with respect to SNP density and deleterious SNP accumulation.

### BCWE and PWCW yeast strains carry genetic variants with relevance to wine fermentation

#### Gene LOF Ontology Enrichment in BCWE, RCW, and PWCW yeast strains

To determine the biological impact of deleterious SNPs in the 4 strain groups, gene LOF was predicted based on the presence of high-impact SNPs and/or variants within coding regions. Here we define high-impact as homozygous variants that cause frameshifts, loss of a start codon, or introduction of a premature stop codon within 98% of the coding region (Bergstrom *et al*. 2014). Among the 102 yeast strains, a total of 493 nondubious S288c genes were predicted to have LOF in at least 1 strain (Supplementary Table 5). Gene ontology (GO) biological process enrichment analysis was performed on LOF genes present in each strain group, but multiple GO enrichment categories were only observed in BCWE and RCW strain groups (Fig. 3d, Supplementary Tables 5a, 5b). No enrichment was observed for the RNAO strain group gene LOF, and only transmembrane transport was enriched for the PWCW strain group (34 genes, Supplementary Table 5c). We, therefore, focused on the biological process enrichment of genes with predicted LOF in the BCWE and RCW strain groups (Fig. 3d). This analysis revealed that transmembrane transport was highly enriched for LOF in BCWE strains (40 genes) and genes involved in carbohydrate transmembrane transport (7 genes) and iron ion transport (8 genes) (Fig. 3d, Supplementary Table 5a). Transmembrane transport was also enriched in RCW strains (31 genes) and genes involved in copper iron import (4 genes), sulfur compound transport (5 genes), and siderophore transport (4 genes) (Fig. 3d, Supplementary Table 5b). Transporter LOF in BCWE and RCW strains could be a mechanism of resistance against toxic compounds or may have arisen due to a lack of selection pressure for transporters of nutrients that are absent in grape juice. The majority of the genes identified with the GO term carbohydrate transmembrane transport were members of the hexose transport (*HXT*) gene family. *HXT1-7* encode transporters with a varying affinity for glucose and fructose, the 2 most abundant sugars in wine grapes, whereas *HXT8-17* have been suggested to have alternative functions, such as polyol sugar transport and pleiotropic drug resistance (Reifenberger *et al*. 1997; Bisson *et al*. 2016; Jordan *et al*. 2016). We find that *HXT* gene LOF occurs in BCWE (*HXT8-11*,*15*,*17*), RCW (*HXT4*,*8*), and PWCW (*HXT1*,*7-9*) strain groups. We also find that genes involved in maltose metabolization, specifically *MAL1* and *MAL3* gene loci and *IMA5*, have LOF in the BCWE and RCW strain groups. The loss of the *MAL* and *IMA* genes likely reflects the absence of maltose and isomaltose in grape must and therefore the lack of need for uptake of these sugars. LOF of *MAL* genes is also observed in 16 PWCW strains but is absent in the RNAO strain group.

Another biological process that is enriched for LOF in both BCWE and RCW strain groups is flocculation (4 genes), suggesting that the ability of some yeasts to adhere to other strains or surfaces may be impaired (Fig. 3d, Supplementary Tables 5a, 5b). Commercial wine yeasts typically exhibit nonflocculant phenotypes as flocculation upon inoculation into a tank full of grape juice results in inefficient and slow or stuck fermentation (Carstens *et al*. 1998; Govender *et al*. 2010). We find that the *FLO* genes associated with cell-cell adhesion—*FLO5*,*9*,*10*,*11*—are enriched for LOF in BCWE and RCW strain groups (Fig. 3d, Supplementary Tables 5a, 5b). *FLO* genes have been previously noted to be maintained in wild populations where cell adherence is likely important but lost in domesticated strains (Duan *et al*. 2018). The exception is a subset of industrial beer strains that have been selected for strong flocculation ability to increase the efficiency of cell separation from beer (Gallone *et al*. 2016). Indeed, we find that none of the RNAO strains contained LOF in *FLO* genes. Despite the fact that we did not identify the GO term “flocculation” enriched in the PWCW strain group, 14 of the PWCW strains do contain high-impact variants in *FLO* genes.

#### Differential gene LOF in RCW, BCWE, PWCW, and RNAO strain groups

**Fig. 4.**
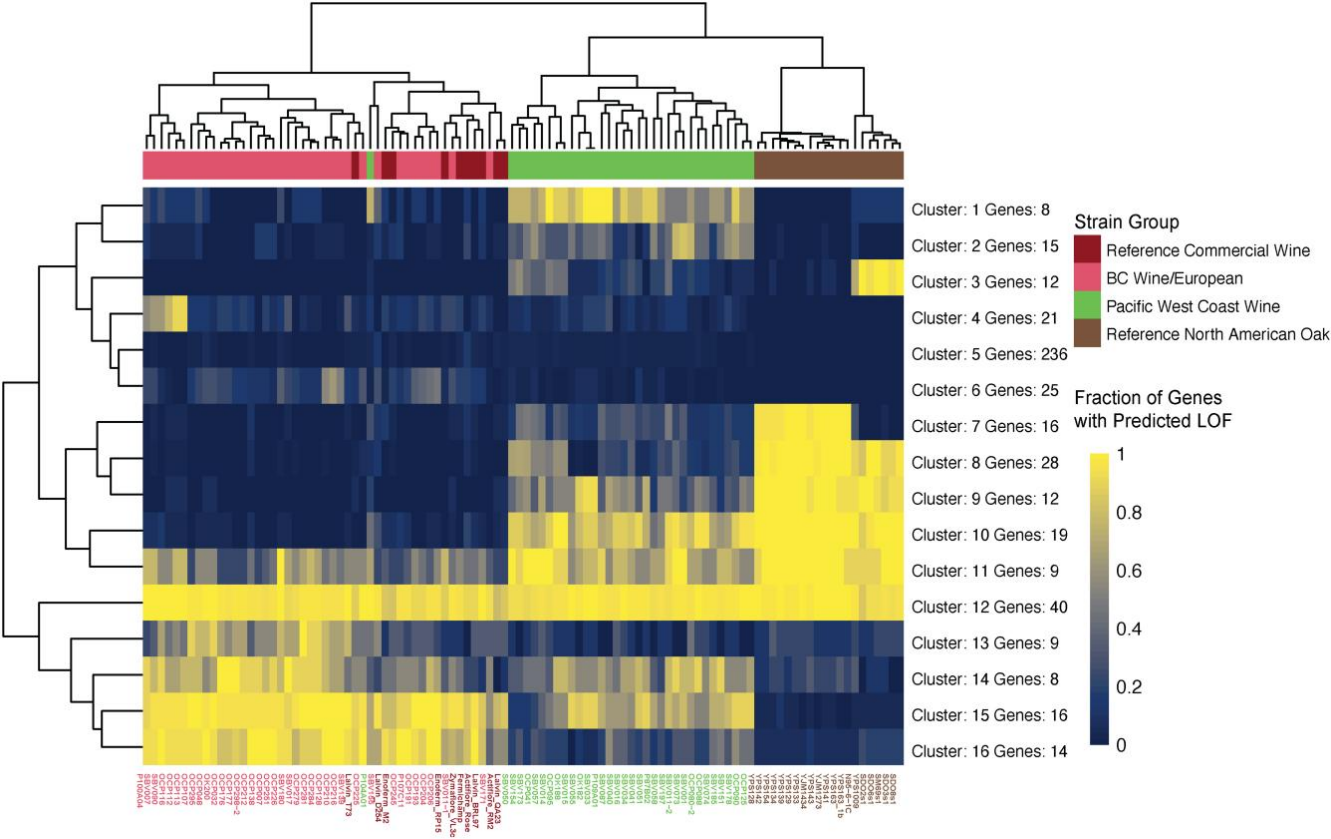
Gene LOF clustering demonstrates allele sharing between the PWCW strain group and each of the RNAO and RCW/BCWE strain groups. *K*-means clustering of genes with predicted LOF, using *k* = 16. Dark blue indicates that a LOF mutation is not present in a given strain; yellow indicates that a LOF mutation is present within a given strain. The range of dark blue to yellow indicates the fraction of genes in each cluster that have LOF mutations per given strain.

We wanted to determine if gene LOF was specifically impacted in strains from the RCW, BCWE, PWCW, and RNAO strain groups. To carry out this analysis, genes predicted to have LOF were clustered together based on their occurrence amongst strains within the 4 groups using a *k*-means approach with *k* = 16. Each cluster contains a group of genes with similar patterns of LOF amongst all strains (Fig. 4). For example, Cluster 16 contains 14 genes with high-impact variants found most frequently in RCW and BCWE strain groups but less frequently in PWCW and RNAO strain groups (Fig. 4, Supplementary Table 6). Cluster 16 includes the *COS12* gene that encodes a yeast multi-vesicular body sorting factor (MacDonald *et al*. 2015). The Cos proteins down-regulate cell surface membrane proteins by sorting them into multivesicular bodies when nutrients are depleted (MacDonald *et al*. 2015). Cluster 13 contains 9 genes with frequent LOF in BCWE strains, 1 of which is the *COS8* gene. Interestingly, a protein-fragment complementation (PFC) screen identified interactions between Cos8 and the Ssu1 plasma membrane sulfite pump and also the Amf1 low-affinity NH4+ transporter (Miller *et al*. 2005). Therefore, the LOF of the *COS* genes could help to stabilize cell surface membrane proteins to transport ammonium when nutrients are low. Stabilization of Ssu1 in BCWE clade strains could help to remove sulfite from the cell after potassium metabisulfite, an antioxidant and antimicrobial, is added to grape juice before fermentation. Clusters 1 and 2 contain 25 genes with frequent LOF specifically in the PWCW strain group (Fig. 4, Supplementary Table 6). We find 22 PWCW strains with LOF in *ZNF1*, a transcription factor involved in nonfermentable carbon utilization and respiratory growth (Tangsombatvichit *et al*. 2015). The loss of functional *ZNF1* in the PWCW strain group may reflect a novel adaptation to the fermentative wine environment. We also find 9 PWCW strains with LOF of *IRC7*, a cysteine desulphydrase involved in the production of aromatic thiol compounds and negative volatile sulfur compounds such as hydrogen sulfide. The inactivation of *IRC7* has been reported to be common in wine yeast strains, and we find predicted LOF of *IRC7* in 5 BCWE strains (Cordente *et al*. 2019).

Clusters 9 and 10 contain genes with frequent high-impact variants in the RNAO and PWCW strain groups that are not frequently found in the RCW or BCWE strain groups (Fig. 4, Supplementary Table 6). We find that Cluster 10 contains the *AQY2* aquaporin that encodes a membrane water channel involved in thermotolerance and freeze-thaw survival. Functional aquaporins are associated with wild yeast strains whereas LOF mutations are found in strains exposed to high osmolarity environments such as grape juice and lab strains (Bonhivers *et al*. 1998; Laize *et al*. 1999; Laize *et al*. 2000; Carbrey *et al*. 2001; Fay *et al*. 2004; Will *et al*. 2010; Clowers *et al*. 2015; Goncalves *et al*. 2016; Pontes *et al*. 2020). *AQY2* has an 11-nucleotide deletion in S288c that inactivates the gene whereas 29 out of 34 PWCW and all 20 RNAO strains retain the 11 nucleotides suggesting that *AQY2* encodes a functional aquaporin in these strains (Laize *et al*. 2000; Carbrey *et al*. 2001; Will *et al*. 2010). In the BCWE strains, however, only 1 out of 38 strains retain the 11 *AQY2* nucleotides and the remaining 37 strains carry the 11-nucleotide deletion. We assessed the full-length *AQY2* gene sequence in a subset of strains that we de-novo assembled and found that indeed strains from the RNAO and PWCW groups contain full-length *AQY2* genes that are identical to functional Σ1278b Aqy2 except for 1 residue (P141S) that has been previously mutated and does not affect osmotic water permeability (Carbrey *et al*. 2001). The maintenance of *AQY2* in the PWCW strains is likely advantageous as the BC vineyard ecosystem experiences freeze-thaw cycles. Clusters 14 and 15 contain genes with frequent LOF in the BCWE and PWCW strain groups, but not frequent LOF in the RNAO strain group (Fig. 4, Supplementary Table 6). We find that Cluster 15 contains *HXT8* and the aquaporin *AQY1* in both BCWE and PWCW strains, which both have relevance to wine domestication.

Differential gene LOF, as observed in this analysis, suggests that there is functional diversity across the 4 strain groups which may result in different fermentative qualities. Furthermore, the clustering of PWCW strains in Fig. 4 suggests that these strains have retained alleles from both RNAO and WE lineages that allow them to adapt to the BC vineyard and winery environment.

### PWCW, BCWE, and RNAO group strains have less genome decay than RCW group strains

**Fig. 5.**
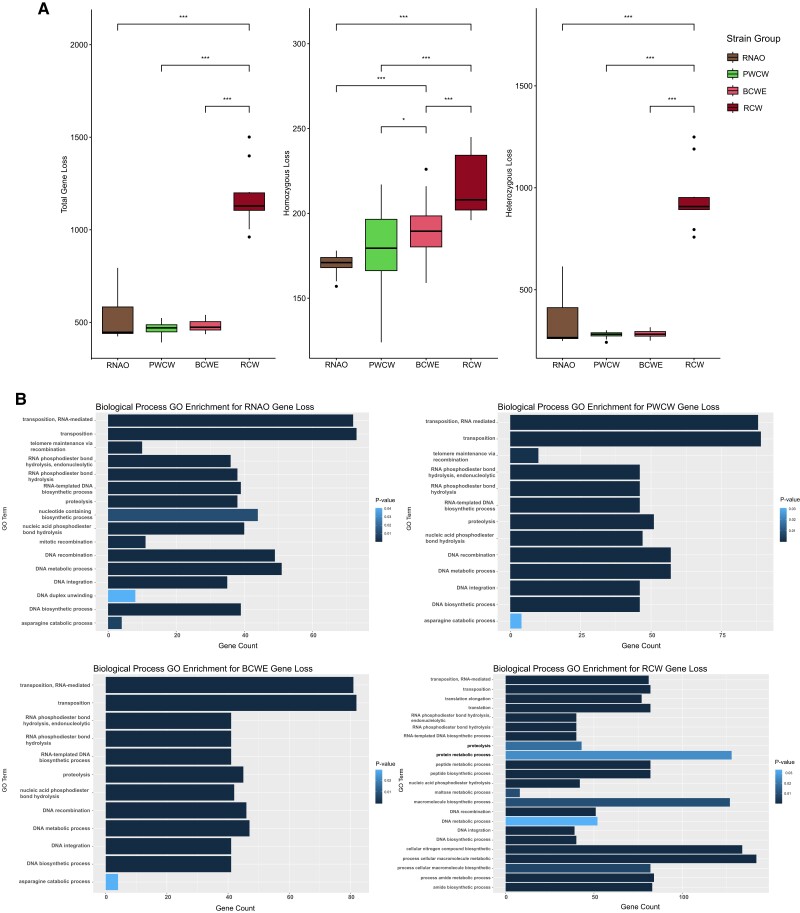
The RCW strain group has increased gene loss when compared to the BCWE, PWCW, and RNAO strain groups. a) Boxplots indicate gene loss events for the 4 diploid strain groups. Total gene loss is considered if gene CN was less than 2. Gene loss is considered homozygous if CN is zero and heterozygous if CN is 1. b) GO Biological Process enrichment analysis of S288c genes that contain a homozygous gene loss in each of the 4 strain groups.

Gene CN loss is a hallmark of microbial domestication (Gibbons *et al*. 2015; Gallone *et al*. 2016; Duan *et al*. 2018; Legras *et al*. 2018; Peter *et al*. 2018). As strains adapt to anthropic environments, new selection pressures and relief from old selection pressures cause genes necessary for survival in the previous habitat to be lost. Recent evidence suggests that gene loss can result in improved fitness phenotypes in a domesticated environment but detrimental fitness in alternative environments (Costanzo *et al*. 2010; Gallone *et al*. 2016; Duan *et al*. 2018; Peter *et al*. 2018; Helsen *et al*. 2020). For this reason, gene content loss was examined as an indicator of previous domestication events in the 4 strain groups previously mentioned with the exception of the YPS163_b haploid strain. Diploid strains were considered to have a gene loss event if the CN for a gene was less than 2 (1 or 0) for a given loci (Supplementary Table 7). We find that both BCWE and PWCW strain groups have less genome decay when compared to the RCW strain group (Fig. 5a). The RCW strain group has a mean of 39 more homozygous deletion events (*P* = 1.0 × 10^−4^) and 675 more heterozygous deletion events than the PWCW strain group (*P* = 6.0 × 10^−6^). In total, the PWCW strain group has a mean of 714 more genes (*P* = 1 × 10^−6^) than the RCW strain group (*P* = 6.2 × 10^−6^) (Fig. 5a). We did not find significant differences in the frequency of total, homozygous or heterozygous gene loss events between the PWCW and RNAO strain groups or between BCWE and RNAO strain groups for total and heterozygous gene loss events (Fig. 5a). The BCWE strain group, however, did have more homozygous gene loss events than both the PWCW (*P* = 0.03) and RNAO (*P* = 1.6 × 10^−5^) strain groups (Fig. 5a). Together these data demonstrate that gene loss is prevalent in commercial strains but is limited in BC strains which may reflect different degrees of domestication. Further, the preservation of genes in the PWCW strain group, even in comparison to the BCWE strain group, supports a requirement for maintaining genomic content for multiple (including potentially nonanthropic) environments.

To understand how gene loss may impact the biological processes associated with the 4 strain groups, we performed GO enrichment analysis (Fig. 5b). This analysis resulted in a total of 11 GO terms shared between all strain groups that all contained genes encoding Ty elements. Therefore, in comparison to the S288c reference genome, all 4 strain groups have fewer Ty elements in their genomes. The high Ty content in lab strains compared to wild strains has been previously observed (Liti *et al*. 2005; Liti *et al*. 2009; Carr *et al*. 2012; Bleykasten-Grosshans *et al*. 2013). We observed that the PWCW and BCWE strain groups contain nearly identical GO terms, except for “telomere maintenance via recombination” which is only enriched in the PWCW and RNAO strain groups and is due to loss of the YRF helicase genes (Y’elements) in the subtelomeric regions (Fig. 5b, Supplementary Table 8). Interestingly, CN depletion of *YRF* genes has been previously noted in wild strains and it has recently been shown that telomeres are shorter in wild *S. cerevisiae* strains compared to domesticated strains (Carreto *et al*. 2008; D’Angiolo *et al*. 2023). By comparison, the RCW strain group has 12 GO terms that are not associated with the other strain groups. These terms, such as translational elongation, translation, and peptide biosynthetic process, are due to the enrichment of tRNA, rRNA, and ribosomal protein genes that are lost in the RCW strain group (Fig. 5b, Supplementary Table 8a).

### Differential CNV analyses suggest wine-making adaptations in the PWCW strain group

**Fig. 6.**
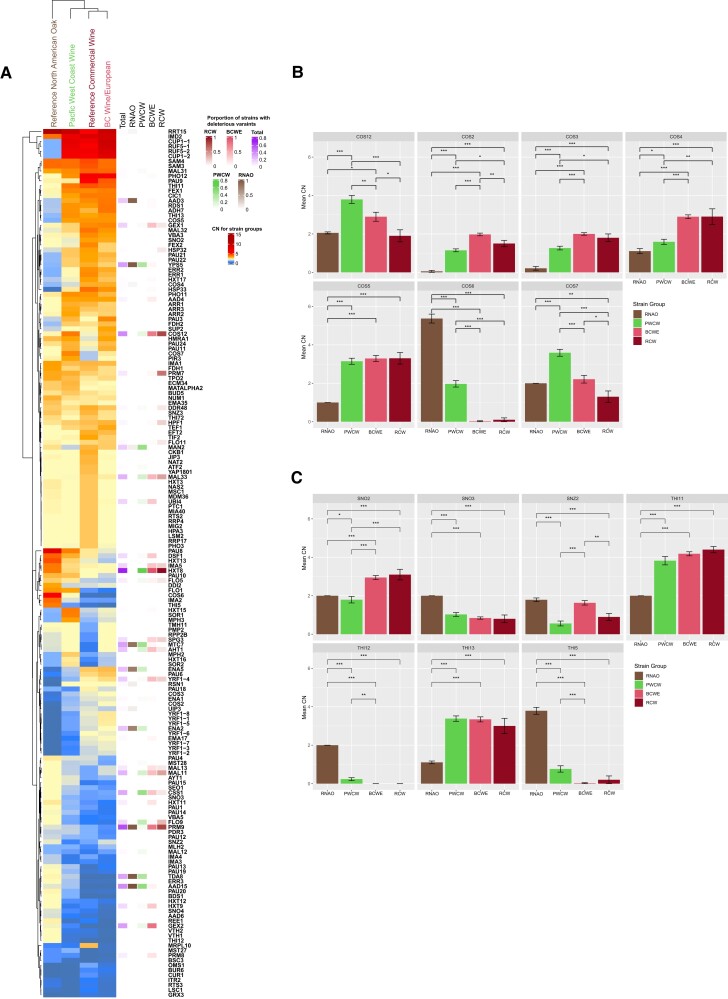
Differential CNV analysis suggests adaptations to wine-making conditions in PWCW strains. a) Heatmap displays significantly differential CNV loci for genes with known functions. Genes with significantly different CNV were identified in the BCWE, PWCW, RCW, and RNAO strain groups. The heatmap displays a subset of 176 genes with known or predicted functions. The values given are the mean CN per strain group with a CN range of 0 (blue) to 15 (dark red). Significant genes were determined using the Kruskal–Wallis test (*P* ≤ 0.05) and a false discovery rate of 0.05. The proportion of strains containing at least 1 high-impact SNP for a given gene is provided for each of the BCWE (red), PWCW (green), RCW (dark red), and RNAO (brown) strain groups. b) Mean CN of *COS2-7,12* genes in BCWE (red), PWCW (green), RCW (dark red), and RNAO (brown) strain groups. c) Mean CN of *SNO2*, *SNO3*, *SNZ2*, *THI11*, *THI12*, *THI3*, and *THI5* in BCWE (red), PWCW (green), RCW (dark red), and RNAO (brown) strain groups. In (b) and (c), strains are diploid, therefore, CN of 2 is expected. In (b) and (c) brackets show results from pairwise Wilcoxon rank sum tests for the strain populations. Significance is indicated by *P ≤ 0.05, **P ≤ 0.01, and ***P ≤ 0.001.

CNV has been suggested as a mechanism for rapid adaptation following environmental stimuli (Jack *et al*. 2015; Hull *et al*. 2017; Lauer *et al*. 2018; Castagnone-Sereno *et al*. 2019). One selective advantage for CNV is a potentially higher phenotypic impact than smaller genomics variants such as SNPs (Peter *et al*. 2018). We tested all diploid strains in the 4 strain groups (BCWE, PWCW, RCW, RNAO) for differential CN in genes as an indicator of potential adaptations to wild or wine-fermentation environments. A Kruskal–Wallace test was employed on the strain CN profile with a false discovery rate (FDR) q-value cut-off of 0.05. This analysis discovered 2,625 genes with significantly differential CN. Genes were further filtered for those most likely to have a strong phenotypic impact by selecting genes that had mean CN values greater than 2.5 or less than 1 for at least 1 of the strain groups. This resulted in 456 genes with differential CN (Supplementary Table 9). As mentioned above, ribosomal RNA (rRNA), tRNA, and Ty element genes have high degrees of CNV and are among the most significant loci discovered in our analysis (19 rDNA genes, 23 tRNA genes, 43 Ty elements, Supplementary Table 9) (Szostak and Wu 1980; Gibbons *et al*. 2015; Jack *et al*. 2015; Steenwyk and Rokas 2017; Peter *et al*. 2018; Salim and Gerton 2019). We chose to focus on 176 protein-coding genes with differential CN that have known or predicted functions and may be relevant to wine-making or life in the vineyard. Of these 176 genes, we were surprised to find that 49 have high-impact mutations with frequencies ranging from 1 to 70 strains. In Fig. 6a we present the differential CN data for 176 genes with known or predicted function and indicate the 49 genes with high-impact mutations, shaded according to the proportion of strains in the strain group that carry the mutation. CN values differing from the expected value of 2 are of particular interest for diploid strains.

We find that the CN profiles of the BCWE strain group cluster with that of the RCW strain group, whereas the PWCW strain group exhibits an intermediate profile between the RCW and RNAO strain group (Fig. 6a). We identified several genes with CNV trends that are distinct to each group of strains which may reflect evolutionary events such as adaptation to the wine-making environment. The use of copper sulfate to treat powdery mildew infection of grape berries has resulted in resistance of wine strains to copper (Mortimer 2000; Fay *et al*. 2004; Warringer *et al*. 2011). It is well documented that CN increase of the *CUP1* gene that encodes the copper-binding metallothionine protein confers resistance to copper sulfate (Fogel *et al*. 1983; Welch *et al*. 1983; Karin *et al*. 1984; Fay *et al*. 2004; Warringer *et al*. 2011; Zhang *et al*. 2013; Zhao *et al*. 2014; Almeida *et al*. 2015; Strope *et al*. 2015; Steenwyk and Rokas 2017; Peter *et al*. 2018; Crosato *et al*. 2020; Yang *et al*. 2018). We also find that the *CUP1-1* and *CUP1-2* copper resistance genes have a mean CN of 5.5 to 9 in the BCWE, RCW, and PWCW strain groups but have a mean CN of 1 in the RNAO strain group (Fig. 6a, Supplementary Table 9). This data suggests that PWCW strains have adapted to life in the vineyard with increased *CUP1* CN compared to the RNAO group strains that have likely not been exposed to copper sulfate.

A study on *S. cerevisiae* wine strain CNV found both amplifications and deletions in the *ADH7* alcohol dehydrogenase and *AAD3* aryl-alcohol dehydrogenase genes (Steenwyk and Rokas 2017). Indeed, genes that function in alcoholic fermentation, including *ADH7*, *AAD3*, and *AAD4*, were found to be amplified in RCW (mean CN of 2.8, 3.5, 3.1, respectively), BCWE (mean CN of 3.9, 3.8, 3.1, respectively) and PWCW (mean CN of 3.3, 2.9, 3.0, respectively) group strains, but not in RNAO (mean CN of 1.3, 1.3, 2.0, respectively) group strains (Fig. 6a, Supplementary Table 9). These observations suggest a role for increased alcoholic dehydrogenase activity, and for a broad range of substrates, which may subsequently improve fermentation performance in RCW, BCWE, and PWCW group strains.

We find differential CN in 7 of the *COS* genes (*COS2-7,12*), a family of 11 genes that are regulators of cell surface membrane protein internalization (Fig. 6b, MacDonald *et al.* 2015). As discussed earlier, the loss or gain of *COS* genes could impact the redistribution of cell surface membrane proteins to the vacuole including amino acid transporters, especially under conditions of nutrient stress (MacDonald *et al*. 2015). Of the 7 *COS* genes with differential CN, we did not find high-impact mutations in the *COS2*, *3*, *5*, *6*, or *7* genes in any of the strain groups suggesting that the differential CN may impact *COS* gene function (Supplementary Table 9, Fig. 6a). We find that the RNAO strain group has a higher mean CN for *COS6* (mean CN of 5.4) compared to the PWCW (mean CN of 2.0), BCWE (mean CN of 0.03), and RCW (mean CN of 0.1) strain groups (Fig. 6b). Notably, a PFC screen identified an interaction between Cos6 and Aqy1; therefore, it would be interesting to determine if there is a connection between freeze-thaw tolerance and *COS6* CN (Miller *et al*. 2005). *COS5* has a mean CN > 3.0 in PWCW, BCWE, and RCW strain groups compared to a mean CN of 1.0 in RNAO (Fig. 6b). Cos5 has an interaction with the Hxt9 hexose transporter which may impact Hxt9 turnover (Miller *et al*. 2005). We also identified *HXT9* as a LOF gene in a subset of BCWE strains (Cluster 6—Fig. 4, Supplementary Tables 6 and 10). In contrast to *COS6*, the *COS2* and *COS3* genes are lost in the RNAO strains (mean CN of 0.1 and 0.2, respectively) and are present in less than 2 copies in all but the BCWE strains (mean CN of 2.0 and 2.0, respectively, Fig. 6b, Supplementary Table 9). There is a PFC interaction between Cos3 and the Thi7 plasmid membrane transporter which could impact thiamin import, which is an important vitamin for wine fermentation as discussed below (Miller *et al*. 2005).

We find differential CN in thiamin (vitamin B1) and pyridoxine (vitamin B6) metabolism genes which do not have high-impact SNPs in the majority of strains analyzed (Fig. 6c, Supplementary Table 9). *THI5*, *THI11*, *THI12*, and *THI13* encode identical enzymes that convert pyridoxal 5′-phosphate (PLP) to 4-amino-5-hydroxymethyl-2-methylpyrimidine phosphate (HMP-P), which is a precursor for thiamin (Perli *et al*. 2020). For the *THI11* gene, we observe ∼4 copies in the RCW (mean CN of 4.4), BCWE (mean CN of 4.2), and PWCW (mean CN of 3.8) strain groups but only 2 copies in the RNAO strain group (mean CN of 2.0, Fig. 6c, Supplementary Table 9). Similarly, the *THI13* has ∼3 copies in the RCW, BCWE, and PWCW strain groups (mean CN of 3.0, 3.3, and 3.4, respectively) but only ∼1 copy in the RNAO (mean CN of 1.1) strain group. Conversely, *THI12* has been lost in all strain groups except the RNAO strain group with a mean CN of 2.0 (Fig. 6c, Supplementary Table 9). *THI5* has reduced CN in the RCW, BCWE, and PWCW strain groups (mean CN of 0.2, 0.0, and 0.8, respectively) but is amplified in RNAO strains (mean CN of 3.8). Thiamin is important for wine fermentations, as it is required for the activity of pyruvate decarboxylase (Pdc), and can be supplemented by winemakers to prevent stuck fermentations (Labuschagne and Divol 2021). Therefore, the increased CN of *THI11* in the RCW, BCWE, and PWCW strain groups suggests a domestication event not present in the RNAO strain group that may regulate thiamin biosynthesis which is energetically taxing on the cell (Perli *et al*. 2020). The *SNO* and *SNZ* genes encode enzymes that catalyze the synthesis of de novo PLP, which in addition to serving as a precursor for thiamin synthesis, is a cofactor for a variety of amino acid biosynthesis enzymes (Perli *et al*. 2020). There are 3 homologous members of each of the *SNO* (*SNO1-3*) and *SNZ* (*SNZ1-3*) genes in the S288c genome. We recorded elevated *SNO2* CN in the RCW (mean CN of 3.1) and BCWE (mean CN of 2.9) strain groups (Fig. 6c, Supplementary Table 9). Only the RNAO strain group has retained both copies of the *SNO3* gene with a mean CN of 2.0 compared to all other strain groups which have a mean CN of 1 or less (Fig. 6c, Supplementary Table 9). The *SNZ2* gene had a mean CN of 1.8 in the RNAO strain group, a mean CN of 1.6 in the BCWE strain group but less than 1 copy in the RCW (mean CN of 0.9) and PWCW (mean CN of 0.6) strain groups (Fig. 6c). The differential CN in thiamin and pyridoxine metabolism genes in the 4 strain groups further supports the hypothesis that the PWCW and BCWE strain groups may have optimized thiamin and pyridoxine biosynthesis as an adaptation to the wine-making environment.

### Pangenome analysis reveals less HGT in BC strains from the PWCW clade

**Fig. 7.**
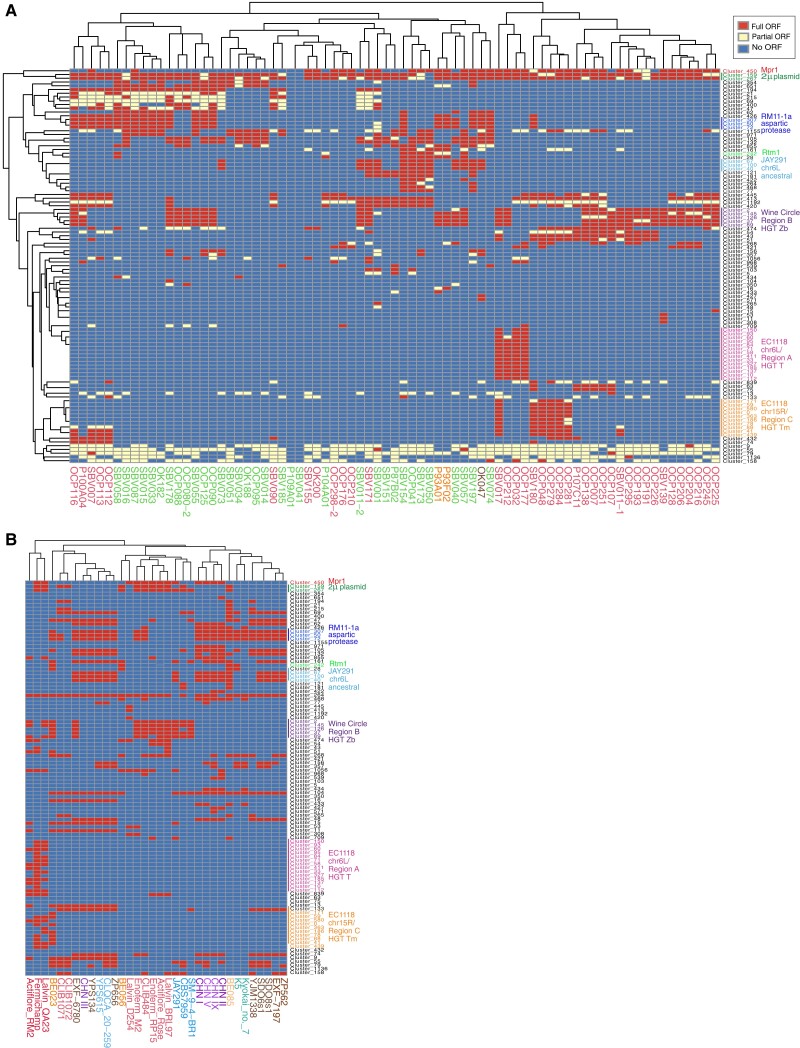
Pan-genomes of BC strains reveal less HGT in BC strains from the PWCW vs WE clades. a) BC strains are indicated on the X-axis and colored according to the clade they belong to in Fig. 1. Each cluster on the Y-axis represents a group of non-S288c ORFs with 97% or higher nucleotide sequence similarity. Red indicates that a full length ORF is present in a given strain, yellow indicates a partial ORF and blue indicates that the ORF is not present. Some of the clusters that have been identified in global strains are highlighted on the right side of the cluster. b) Non-S288c clusters from (a) were compared to non-S288c ORFs predicted from 34 global strains to determine which gene clusters are present in the global strain genomes. Global strains are colored according to which clade they belong to in Fig. 1.

The *S. cerevisiae* pangenome contains 7,796 ORFs compared to the 6,081 nonredundant ORFs in the S288c reference genome (Strope *et al*. 2015; McIlwain *et al*. 2016; Duan *et al*. 2018; Legras *et al*. 2018; Peter *et al*. 2018). The 1,715 ORFS not represented in the S288c genome, which we will refer to as “non-S288c genes” are either part of the ancestral *S. cerevisiae* genome or have been acquired by HGT from other yeast species or by introgression from mating with *Saccharomyces species* (Duan *et al*. 2018; Legras *et al*. 2018; Peter *et al*. 2018). In particular, commercial wine strains contain a cluster of 5 genes horizontally transferred from *Z. bailii*, referred to as “the wine circle/Region B” which is proposed to provide an advantage during wine fermentation (Borneman *et al*. 2011; Galeote *et al*. 2011). To determine which non-S288c genes are present in the 75 BC strains, after mapping to the S288c genome, we de novo assembled all remaining reads, and predicted ORFs, followed by clustering to identify ORFs with 97% or more nucleotide similarity to each other. The clusters were compared against 2 pan-genomes and manually curated (Fig. 7a, Supplementary Table 11, see Materials and Methods). We chose a subset of global strains to assess for the presence or absence of the non-S288c genes present in the BC strains. The global strains included commercial wine strains from the WE clade and strains from the CHNI, II, III, V, IX, Beer 1/Mixed Origin, Beer 2, Brazilian Bioethanol, Ecuadorian, Mediterranean Oak, Sake, and TPO clades. Due to the difference in sequencing read depth and coverage we did not co-cluster global strain non-S288c genes with our BC strain non-S288c genes. Instead, for our subset of global strains we de novo assembled the genomes, predicted ORFs, then did a BLASTn against our non-S288c clusters from Fig. 7a at 99% query coverage and 97% identity. The resultant heatmap demonstrates which gene clusters are present in the subset of global strains (Fig. 7b).

We find that the wine circle/Region B is present in 32 out of 75 BC strains (43%), the majority of which are the WE clade strains (21/38 or 55%), as previously noticed for commercial wine strains (Fig. 7a,b) (Borneman *et al*. 2011; Galeote *et al*. 2011; Borneman *et al*. 2016; Legras *et al*. 2018). Both of the BC strains in the Beer 1/Mixed Origin clade (P93A01 and P93F02) contain the wine circle/Region B, but only 9/34 (26%) of the PWCW strains. The single BC strain in the TPO clade, OK047, does not carry the wine circle/Region B (Fig. 7a). Two regions of the industrial wine yeast EC1118 genome, Region A which is an HGT from a *Torulaspora* species and Region C, which is an HGT from *Torulaspora microellipsoides*, are found in a subset of BC strains from the WE clade but are not present in any other BC strains (Fig. 7a,b) (Novo *et al*. 2009). Interestingly, 3 genes (Clusters 67, 100, 40) identified in the JAY291 and GLBRCY22-3 bioethanol strains, a low-affinity NH4+ transporter (*AMF71*), a Zinc finger transcription factor (*ZTF2*) and a siderophore iron transporter with similarity to S288c *SIT1* and *ARN1* were identified in the TPO clade strain OK047 and 29% (10/34) of the BC strains in the PWCW clade but only in 1 WE clade strain (SBV171) and in neither of the BC strains in the Beer 1/Mixed Origin clade (Fig. 7a) (Argueso *et al*. 2009; McIlwain *et al*. 2016). These 3 JAY291 genes are ancestral to the *S. cerevisiae* genome as they are in the wild CHN strains isolated from primeval forests in China (Fig. 7b; Duan *et al*. 2018). The resistance to molasses (*RTM1*) gene which is commonly found in bioethanol, distillery, and beer brewing strains but rarely in commercial wine strains was identified in 8 of our BC strains (Ness and Aigle 1995; Denayrolles *et al*. 1997; Borneman *et al*. 2016; McIlwain *et al*. 2016; Pontes *et al*. 2020). We find full-length *RTM1* in 6 BC strains from the PWCW clade, with partial length *RTM1* in both BC strains from the Beer 1/Mixed Origin clade and no *RTM1* in the BC strains from the WE clade (Fig. 7a). *RTM1* is not found in the ancestral CHN strains (CHNIX, I, II, III, and V, Fig. 7b); however, *RTM1* is present in both domesticated and wild strains isolated from tropical environments and in high sugar environments (e.g. Malaysia and the Philippines) (Pontes *et al*. 2020). *RTM1* was not identified in the TPO clade or WE clade strains that we analysed, however, it was identified in strains BE023 and BE056 (Beer 1/Mixed Origin clade) and BE085 (Beer 2 clade) (Fig. 7b). Given the similarity between the PWCW clade and both beer clades based on Fst metrics (Fig. 2a) and that 2 BC strains from the Beer 1/Mixed Origin clade with partial *RTM1* were isolated in this study, *RTM1* may have been introduced into the PWCW clade through gene flow from beer strains.

When we compared our non-S288c ORF clusters against 2 pan-genome studies, we noticed that a number of clusters had the same S288c homolog, and the same match to 2 pan-genome studies (Borneman *et al*. 2016; Peter *et al*. 2018). For example, clusters 9, 11, 13, 15, 16, and 21 all identified haze protection factor 1 (Hpf1/YOL155C), a secreted cell wall glycoprotein, as the closest S288c homolog and K7 Awa1 from a pan-genome study (Borneman *et al*. 2016). The *AWA1* gene was identified in the Kyokai No. 7 sake yeast strain and is a cell wall protein that is required for foam-formation and cell surface hydrophobicity of sake yeast during sake fermentation (Shimoi *et al*. 2002). The Awa1 protein is 1,713 amino acids and shares significant similarities with the 967 amino acid Hpf1 protein, including the N-terminal signal peptide sequence, a serine-rich sequence, C-terminal repeat sequences, and a glycosylphosphatidylinositol (GPI) anchor domain to target Awa1 to the plasma membrane followed by incorporation into the cell wall (Shimoi *et al*. 2002). We aligned Hpf1 with clusters 9, 11, 13, 15, 16, and 21 and also included the S288c Css1/YIL169C protein which has 66% identity with Hpf1 (Supplementary Fig. 4). We noticed that the N-terminal 37 amino acids that contain a putative signal peptide were highly similar between Hpf1, Css1, and all 6 clusters (Supplementary Fig. 4). The Hpf1 GPI anchor domain at the C-terminal 26 amino acids are conserved between Hpf1 and all of the clusters but is not conserved in Css1 (Supplementary Fig. 4). We used an on-line GPI-anchor prediction site to confirm that Awa1, Hpf1 and all clusters have highly probable GPI-anchors but Css1 does not (Pierleoni *et al*. 2008). There is also a region of high identity amongst all proteins from Hfp1 amino acids 298 to 953 which contains Ser/Thr repeat sequences characteristic of yeast cell wall proteins (Orlean 2012). Cluster 21 is an introgression from *S. paradoxus* and along with clusters 9 and 13 is present in multiple BC and global strain genomes. However, cluster 16 is only present in the 2 Beer 1/Mixed Origin clade strain genomes (P93A01 and P93F02) whereas cluster 11 is only present in the WE clade strain SBV139 and cluster 15 is only present in WE clade strain OCP225. Our data suggest that there may be a large family of Hpf1 homologs in different *S. cerevisiae* strain genomes, however, further proof is required including long-read sequencing and mRNA expression data. Based on our ORF cluster data, other S288c genes that may have multiple family members include the *UIP3* integral membrane protein (5 clusters), the *DIP5* permease (3 clusters), the *ATO3* ammonium transporter (3 clusters) and *MST27/28*, *PRM8/9* integral membrane proteins (7 clusters) (Supplementary Table 11).

## Discussion

### Microsatellites as a predictor of whole genome diversity

We chose a diverse set of 75 *S. cerevisiae* strains isolated from spontaneous grape fermentations for WGS based on microsatellite analysis (Supplementary Tables 1 and 2). Microsatellite analysis is a rapid and inexpensive method to differentiate strains isolated from spontaneous fermentations. However, the resolving power of microsatellites is clearly limited to the number of loci analyzed and whether or not this method is sufficient to determine the genetic relatedness of strains has not been clear. Our WGS analysis demonstrates that the 75 BC yeast strains fall into 4 clades: WE (38 strains), PWCW (34 strains), Beer 1/Mixed Origin (2 strains), and TPO (1 strain) (Fig. 1, Supplementary Table 3). We find that microsatellites are an excellent predictor of genetic relatedness as the WE and Beer 1/Mixed Origin strains are clearly separated from the TPO and PWCW strains (Supplementary Fig. 1). We also find that the microsatellite profiles of the WE and Beer 1/Mixed Origin clade strains exhibit increased heterozygosity, which may serve as another marker for identifying domesticated strains in the absence of WGS (Supplementary Table 2). Analysis of 106 conserved loci from New Zealand *S. cerevisiae* populations isolated from spontaneous wine fermentations revealed their origin to be from the WE clade, despite microsatellite data suggesting distinct populations (Goddard *et al*. 2010; Knight and Goddard 2015; Gayevskiy *et al*. 2016). However, when whole genomes of *S. cerevisiae* strains isolated from New Zealand wine regions were sequenced, it was found that New Zealand strains are a recently diverged population from the European wine clade with genomic signatures of adaptation to the New Zealand habitat (Higgins *et al*. 2021).

### Evolution of the PWCW clade from North American Oak strains

Our analysis of population structure and genetics suggests that the evolutionary history of the PWCW clade is complex. The phylogeny of PWCW strains indicates that the clade is genetically distant from WE clade strains with much greater nucleotide diversity (Fig. 1, Supplementary Fig. 2). Our admixture analysis also found that at *K* = 34, PWCW clade strains share population structure with strains from the TPO clade, specifically North Carolina oak strains and a cluster of mosaic strains (Fig. 1). F-branch statistics and a genome-wide Fst pairwise comparison of population differentiation, however, identified gene flow of domesticated WE strains and wild Ecuadorian strains into PWCW strains (Fig. 2, Supplementary Fig. 3). This analysis leads us to propose that PWCW strains evolved from TPO strains via migration from their oak habitat to a vineyard ecosystem. This migration resulted in the acquisition of genes relevant to survival on the vineyard and in wine fermentation through gene flow from WE strains, which likely is the fastest route of adaptation. Within vineyards, there is evidence of migration between grapes and oak trees as strains isolated from vineyard grapes have clustered with North American oak lineages (Hyma and Fay 2013). Previous studies have similarly reported mosaics of wild and domesticated strains with North American cherry tree orchard and Brazilian rum strains (Cromie *et al*. 2013; Hyma and Fay 2013; Clowers *et al*. 2015; Legras *et al*. 2018). The introgression of Ecuadorian alleles into PWCW strains suggests that Ecuadorian strains have migrated into North America. This hypothesis is supported as 30% of the Ecuadorian strains in our analysis were isolated from trees in the USA, and f-branch statistics support additional gene flow between the Ecuadorian and TPO clades (Supplementary Fig. 3) (Peter *et al*. 2018).

### Gene LOF and CNV suggest partial domestication of the PWCW clade

Industrial strains that have adapted to a specific niche have genome signatures suggestive of domestication (Steensels *et al*. 2019). These domestication hallmarks include low sequence diversity, high rates of heterozygosity, and genome decay that suggests niche specialization. The BCWE and RCW strain groups strongly fit this definition of domestication as they have heterozygous genomes with low sequence diversity, deleterious SNP accumulation, and gene loss (Figs. 3a,c, 5a). In contrast, we find that nearly all PWCW strains contain homozygous genomes and are diploid with no chromosome aneuploidy, suggesting that these strains have lifestyle traits similar to wild oak strains and are efficient at sporulation (Fig. 3a). Sporulation ability may have been maintained in PWCW strains to survive periods of nutrient scarcity in the vineyard. We also find that both the PWCW and RNAO strain groups are enriched for gene loss of subtelomeric Y-elements, suggesting shorter telomeres when compared to the BCWE and RCW strain groups (Fig. 5b, Supplementary Table 8). It has recently been shown that wild and feral strains contain shorter telomeres than domesticated strains, which is hypothesized to be an adaptation to survive conditions in the wild (D’Angiolo *et al*. 2023). When assessing total SNPs and the percent of deleterious SNPs, however, we find that the PWCW strain group lies between the RNAO and BCWE/RCW strain groups suggesting partial domestication (Fig. 3b,c). Similarly, when we analyzed patterns of genes with high-impact SNPs (predicted LOF) in the different strain groups, we find that the PWCW strains share alleles with both the BCWE/RCW and RNAO strain groups (Fig. 4, Supplementary Table 6). Indeed, many such alleles suggest that the PWCW genomes have been selected for both the natural vineyard habitat (*AQY2*) and the anthropic fermentative wine environment (*IRC7*, *FLO*, *AQY1*). The presence of functional *AQY2* but nonfunctional *AQY1* in PWCW strains may reflect a balancing act between surviving freeze-thaw cycles in the Okanagan Valley and surviving high osmolarity during wine fermentation. Hybrids of oak and vineyard strains isolated from North American cherry trees experience similar selection, with some strains containing heterozygous functional *AQY2* alleles (Clowers *et al*. 2015). The PWCW strain group also clusters with the RCW and BCWE strain groups in the differential CNV analysis suggesting that the PWCW strains may have rapidly acquired CNV in genes that are advantageous to the wine-making environment (Fig. 6a). For example, the *THI* genes, that are needed for thiamin synthesis, have similar CN levels in PWCW, RCW and BCWE strain groups (Fig. 6c). As thiamin is required for wine fermentation, we interpret this data to suggest that the *THI* gene CN levels in PWCW, RCW, and BCWE strains are optimized for wine fermentation. However, it remains to be determined if strains in the PWCW clade can compete with strains in the WE clade in wine fermentations. It is notable, however, that 31 of the 34 BC strains in the PWCW clade were isolated from late-stage fermentations with only 3 strains isolated from early-stage fermentations suggesting that the majority of PWCW strains can persist throughout a wine fermentation. As BCWE strains were also isolated from both BC vineyard and winery fermentations, it is likely that BCWE strains also have adaptations for survival in the vineyard environment, albeit with different alleles than PWCW strains. The relative fitness of PWCW vs BCWE strains in vineyard conditions and their ability to persist in the vineyard through periods of nutrient deprivation and freeze-thaw stress in multiple vintages would be interesting to evaluate.

### Limited HGT in PWCW strains supports partial domestication

Investigation of the non-S288c genome content reveals that the PWCW clade strains do not share commonly acquired regions found in commercial wine strains such as EC1118 Regions A and C (Fig. 7a,b). Other larger-scale yeast population studies have found that EC1118 Regions A, B, and C are generally absent in wild strains but widespread in strains isolated from liquid state fermentations such as beer, sherry, and wine (Legras *et al*. 2018; Han *et al*. 2021). Of the 75 BC strains that we isolated from spontaneous wine fermentations, we find that the “wine circle/Region B” genes are present in 55% of the BCWE clade strains and 26% of the PWCW clade strains (Fig. 7a). Notably, the wine circle/Region B is not present in the BC TPO clade strain OK047 nor in the wild North Carolina oak strains that OK047 clusters with (Fig. 7). HGT occurs frequently in fermentation environments and therefore the transfer of the “wine circle/Region B” in the PWCW clade strains supports gene flow from WE clade strains (Steensels *et al*. 2019).

Strains isolated from both grapes and oak trees in North American vineyards have been shown to cluster with either WE clade strains or North American oak strains suggesting that North American vineyards harbor both a native yeast population and a European wine yeast population (Hyma and Fay 2013). Although the majority of PWCW strains were isolated from late-stage spontaneous fermentations, they were isolated from a mixed population and therefore we do not know yet if these strains can thrive in wine fermentations as single-culture inoculums. However, if the PWCW strains with no evidence of HGT can complete wine fermentations as monocultures, it suggests that these strains have acquired other genomic features allowing them to thrive in a wine environment. Our analysis of the pan-genome in BC strains suggests that multiple non-S288c genes are members of larger gene families than previously thought. For example, there may be as many as 6 members of the Hpf1 protein family, in addition to S288c Hpf1 and Css1 in our BC strains. Interestingly, Hpf1 and an Hpf1-like protein have recently been shown to control the chronological life span of *S. cerevisiae* (Barre *et al*. 2020; De Chiara *et al*. 2022). This study reveals that strains isolated from spontaneous wine fermentations are more diverse than previously realized and suggests that isolation and sequencing of *S. cerevisiae* strains from all global wine regions is necessary to fully understand how this species has evolved to withstand the stress of wine fermentation.

## Supplementary Material

jkad130_Supplementary_Data

## Data Availability

The *S. cerevisiae* BC wine strain sequencing data from this work are uploaded to NCBI Sequence Read Archive (SRA) data and are available as BioProject ID PRJNA838724. VCF files are available at figshare: https://doi.org/10.25387/g3.23290001. Supplemental material available at G3 online.
