# Supplementary material for: Whole genome sequencing of Canadian Saccharomyces cerevisiae strains isolated from spontaneous wine fermentations reveals a new Pacific West Coast Wine clade
Source: G3 (Bethesda). 2023 Jun 12;13(8):jkad130. doi: 10.1093/g3journal/jkad130 (PMC10411583; doi:10.1093/g3journal/jkad130)
Supplement: jkad130_Supplementary_Data [file jkad130_supplementary_data.zip › Figure_S4_G3-2023-404213.pdf]

Reference sequence (1): CSS1/YIL169C
Identities normalised by aligned length.
Colored by: property

Figure S4

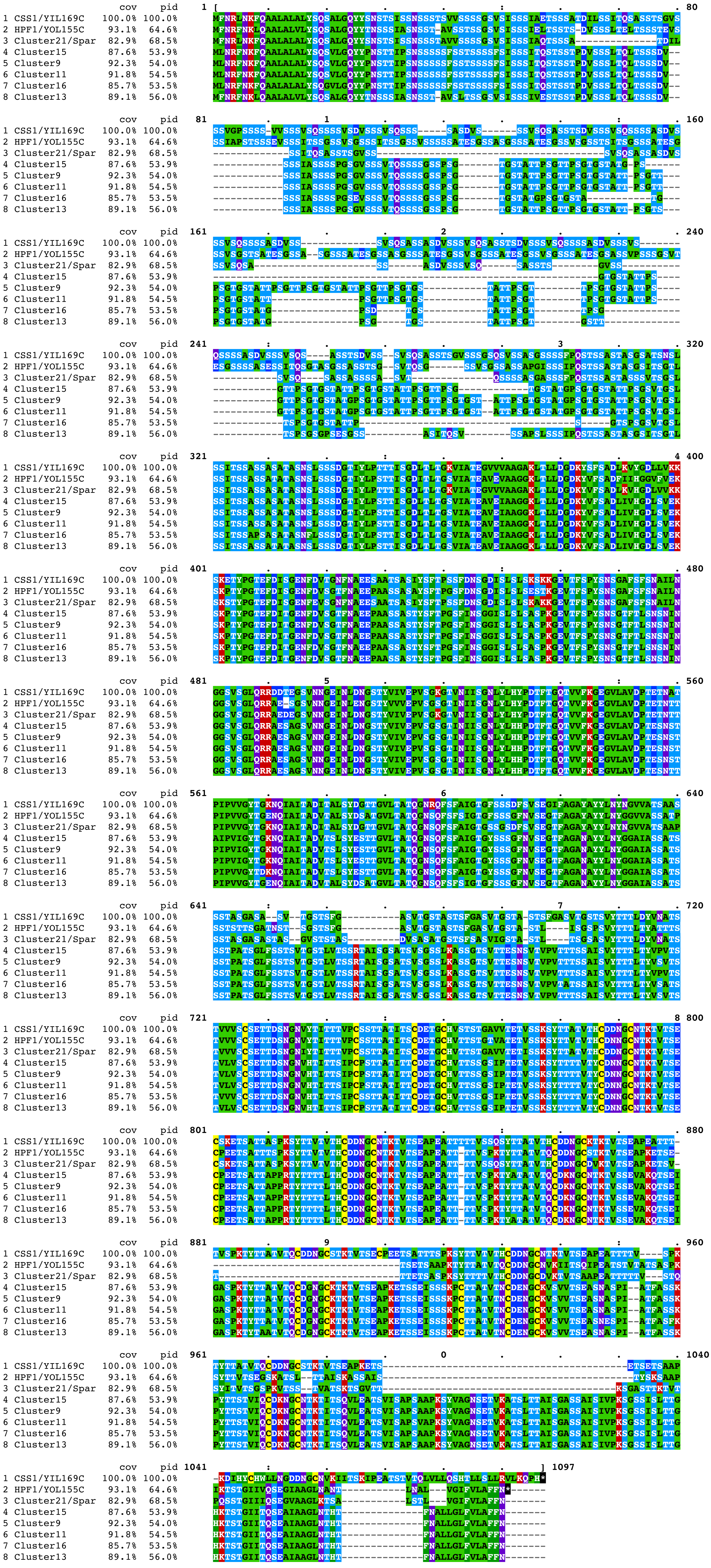

MView 1.63, Copyright © 1997-2018 Nigel P. Brown
