# Supplementary material for: Whole genome sequencing of Canadian Saccharomyces cerevisiae strains isolated from spontaneous wine fermentations reveals a new Pacific West Coast Wine clade
Source: G3 (Bethesda). 2023 Jun 12;13(8):jkad130. doi: 10.1093/g3journal/jkad130 (PMC10411583; doi:10.1093/g3journal/jkad130)
Supplement: jkad130_Supplementary_Data [file jkad130_supplementary_data.zip › Supplemental_Figure_Legends_G3-2023-404213.pdf]

Whole Genome Sequencing of Canadian *Saccharomyces cerevisiae* strains isolated from spontaneous wine fermentations reveals a new Pacific West Coast Wine Clade

Supplemental Figure legends

Figure S1. An unrooted phylogenetic network of 10 commercial wine and 75 BC *S. cerevisiae* strain microsatellite profiles. The network was computed from a matrix of microsatellite-derived Bruvo distance values using the NeighborNet algorithm. WGS clades are denoted by colour: WE (light red), Prise de Mousse/Vin7 (dark red), TPO (brown), PWCW (green) and Beer 1/Mixed Origin (orange). Microsatellite phylogenetic networks were computed in SplitsTree 4.19 (Huson and Bryant 2006) using a Bruvo Distance matrix of microsatellite profiles (Bruvo et al. 2004).

Figure S2. Maximum likelihood phylogenetic tree of 75 BC isolates and 296 *S. cerevisiae* global isolates with branch length. Maximum-likelihood phylogenetic tree of 371 *S. cerevisiae* isolates with *S. paradoxus* strain CBS432 as an outgroup using 477,158 genome-wide SNPs with branch length. Branches are colored based on defined clades/lineages in Figure 1 and are represented in the legend.

Figure S3. F-branch statistic heatmap matrix for indication of gene-flow. F-branch statistic heatmap matrix using 477,158 genome-wide SNPs, for indication of gene-flow estimated from Dsuite based on a collapsed clade phylogenic tree. The input clade phylogenetic tree is represented on the X-axis and the expanded input phylogenetic clade tree is represented on the Y-axis, where dotted lines are internal branches. Higher f-branch values within the matrix suggest excess allele sharing between the branch on the Y-axis (in relation to its sister branch) to the population on the X-axis.

Figure S4. Multiple sequence alignment of Hpf1 (S288c) and 6 pangenome clusters suggest a large family of Hpf1 homologues in the *S. cerevisiae* pangenome. Clustal Omega was used to perform multiple sequence alignment (MSA) on the amino acid sequences of Hpf1(S288c), 6 protein clusters identified in our pangenome analysis, and Css1(S288c), which shares 65% identity with Hpf1 (Sievers et al. 2011). MView was used to visualize the MSA (Brown et al. 1998). Residues are colour based on physiochemical properties, with hydrophobic, polar uncharged, positively-charged, and alcohol amino acids coloured in light/dark green, purple, red, and light/dark blue, respectively. Cysteines are coloured in yellow. Percent coverage and percent identity is relative to Hpf1, and is indicated by "cov" and "pid", respectively.

References

Brown, N.P., C. Leroy, and C. Sander, 1998 MView: a web-compatible database search or multiple alignment viewer. *Bioinformatics* 14 (4):380-381.

Bruvo, R., N.K. Michiels, T.G. D'Souza, and H. Schulenburg, 2004 A simple method for the calculation of microsatellite genotype distances irrespective of ploidy level. *Mol Ecol* 13 (7):2101-2106.

Huson, D.H., and D. Bryant, 2006 Application of phylogenetic networks in evolutionary studies. *Mol Biol Evol* 23 (2):254-267.

Sievers, F., A. Wilm, D. Dineen, T.J. Gibson, K. Karplus *et al.*, 2011 Fast, scalable generation of high-quality protein multiple sequence alignments using Clustal Omega. *Mol Syst Biol* 7:539.
