# Supplementary figures and images for: Whole genome sequencing of Canadian Saccharomyces cerevisiae strains isolated from spontaneous wine fermentations reveals a new Pacific West Coast Wine clade
Source: G3 (Bethesda). 2023 Jun 12;13(8):jkad130. doi: 10.1093/g3journal/jkad130 (PMC10411583; doi:10.1093/g3journal/jkad130)

Figure S1.

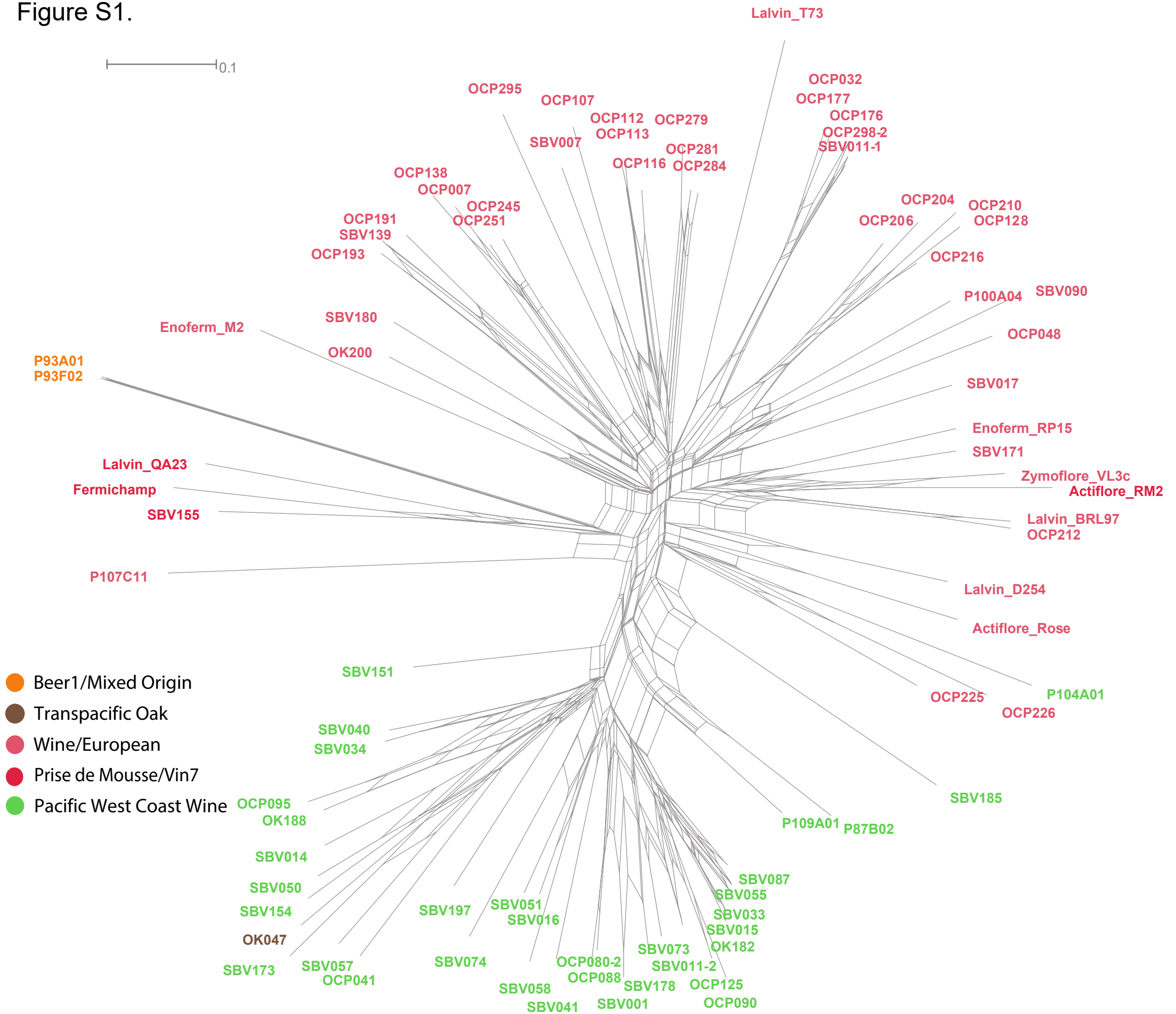

Supplement: jkad130_Supplementary_Data [file jkad130_supplementary_data.zip › Figure_S1_G3-2023-404213.pdf]

Figure S2.

Tree scale: 0.01

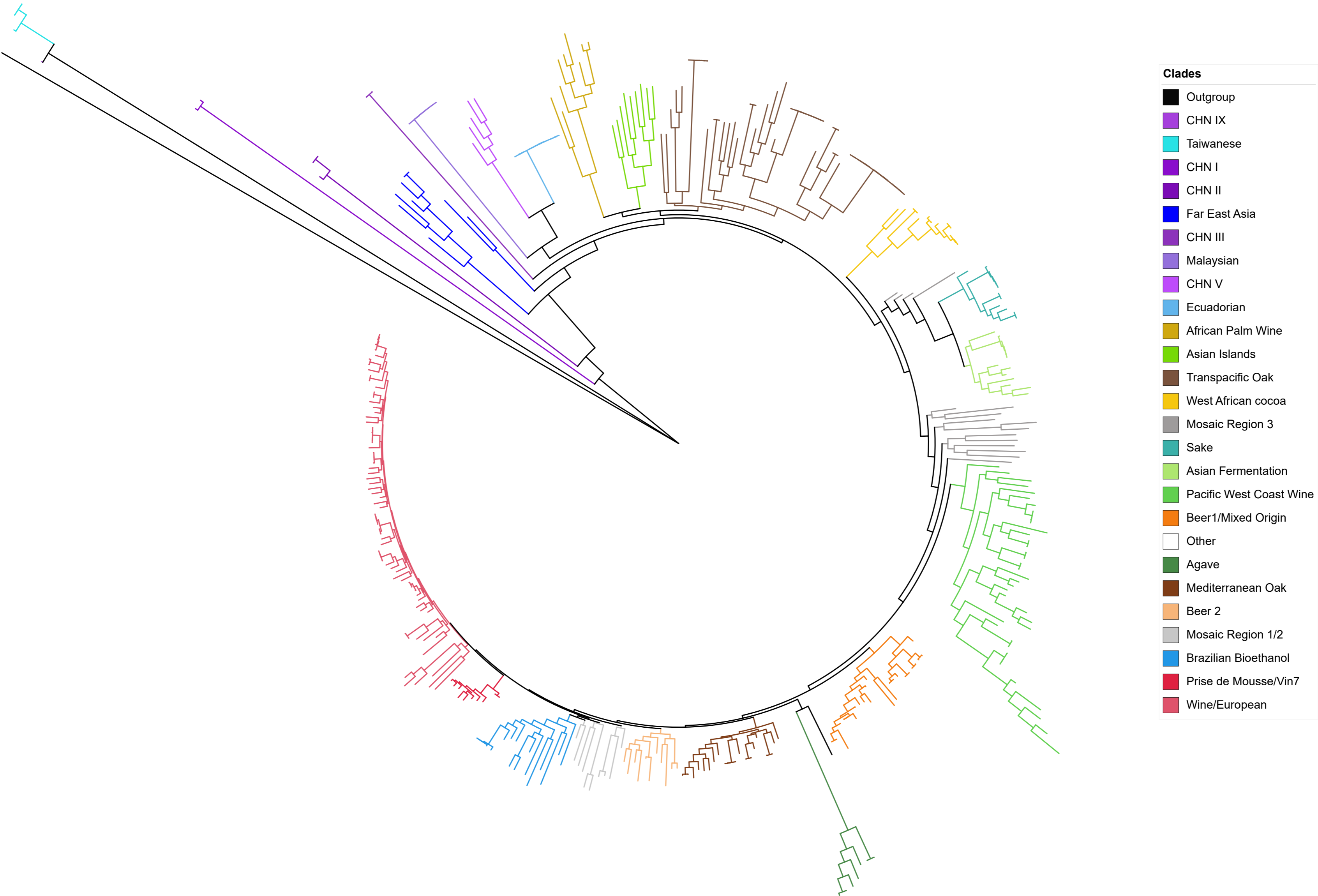

Supplement: jkad130_Supplementary_Data [file jkad130_supplementary_data.zip › Figure_S2_G3-2023-404213.pdf]

Figure S3

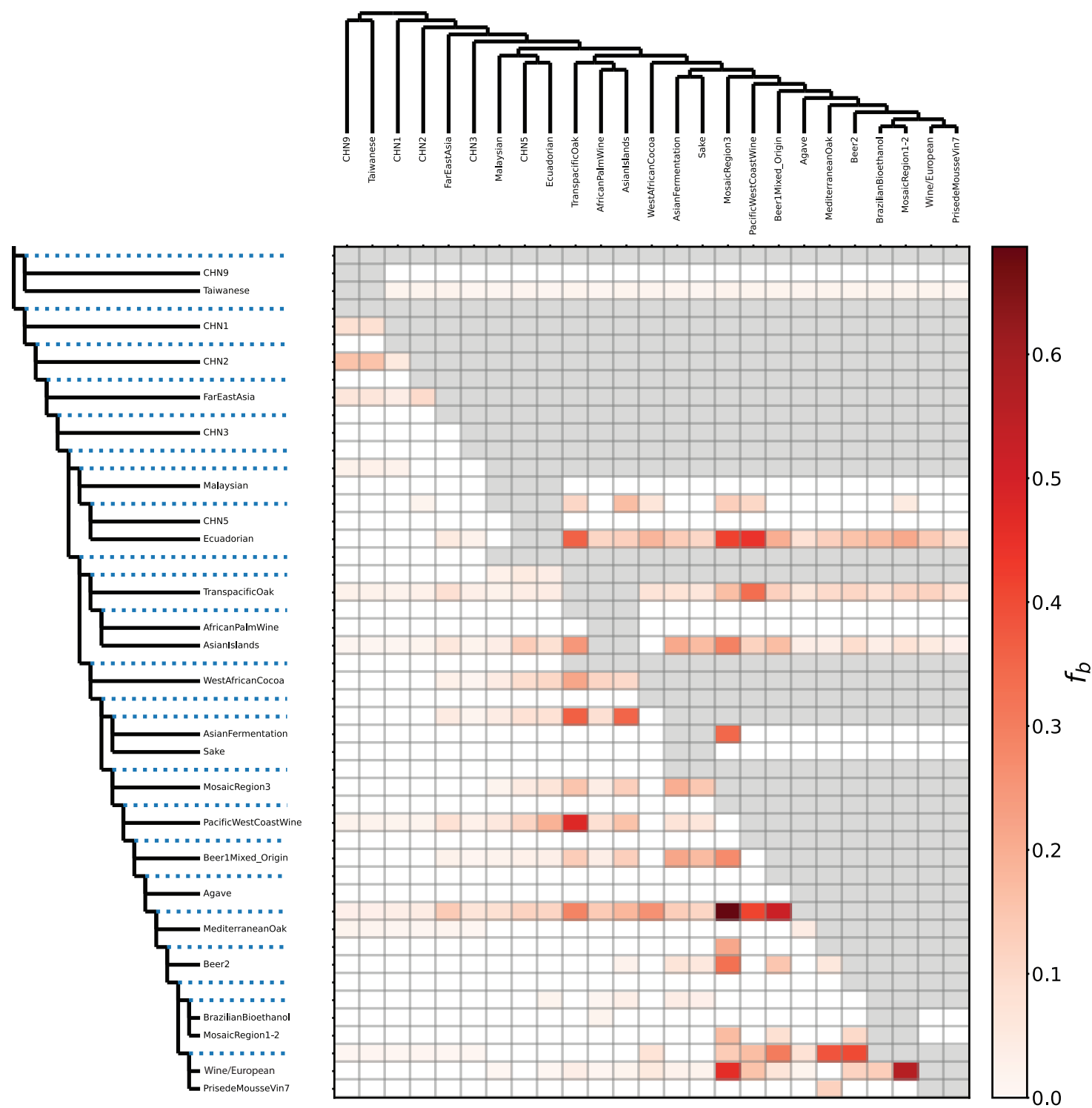

Supplement: jkad130_Supplementary_Data [file jkad130_supplementary_data.zip › Figure_S3_G3-2023-404213.pdf]
